# Epigenetic Biomarkers Driven by Environmental Toxins Associated with Alzheimer’s Disease, Parkinson’s Disease, and Amyotrophic Lateral Sclerosis in the United States: A Systematic Review

**DOI:** 10.3390/toxics13020114

**Published:** 2025-01-31

**Authors:** Melanie Engstrom Newell, Anumitha Aravindan, Ayesha Babbrah, Rolf U. Halden

**Affiliations:** 1 Biodesign Institute, Arizona State University, Tempe, AZ 85287, USA; 2 Biodesign Center for Environmental Health Engineering, Tempe, AZ 85287, USA; 3 School for Engineering of Matter, Transport and Energy, Arizona State University, Tempe, AZ 85287, USA; 4 Barrett, The Honors College, Arizona State University, Tempe, AZ 85287, USA; 5 School of Sustainable Engineering and The Built Environment, Arizona State University, Tempe, AZ 85287, USA

**Keywords:** neurodegenerative disease, environmental factor, biomarker, epigenetic, geography, diagnosis

## Abstract

Environmental toxins and epigenetic changes have been linked to neurodegenerative diseases, including Alzheimer’s Disease (AD), Parkinson’s Disease (PD), and amyotrophic lateral sclerosis (ALS). This paper aimed to (i) identify environmental toxins associated with AD, PD, and ALS, (ii) locate potential industrial sources of toxins in the United States (U.S.), and (iii) assess epigenetic changes driven by exposure to toxins reported by patients. Environmental factors and epigenetic biomarkers of neurodegeneration were compiled from 69 studies in the literature using Preferred Reporting Items for Systematic Reviews and Meta Analyses (PRISMA) and geographic information system approaches. Some 127 environmental toxins have been associated or putatively associated with AD, PD, or ALS, with four toxic metals (As, Cd, Mn, and Hg) common to all three of these neurodegenerative diseases. Environmental toxins associated with epigenetic changes (e.g., DNA methylation) in patients include air pollutants, metals, and organic chemicals (e.g., pesticides, mycotoxins, and cyanotoxins). Geographic analysis showed that study locations (e.g., U.S., Europe, and East Asia) were selected by researchers based on convenience of access rather than exposure risk and disease prevalence. We conclude that several toxins and epigenetic markers shared among neurodegenerative diseases could serve as attractive future targets guiding environmental quality improvements and aiding in early disease detection.

## 1. Introduction

Exposure to environmental factors such as heavy metals over time is known to lead to more frequent and more rapid neurodegeneration, triggering disease development and progression [1,2]. While environmental exposures are widely known to drive disease, the translation of this knowledge to diagnosis and treatment has been limited [3]. Scientific debate over association versus causality and the best methods to screen for exposure to toxins associated with disease continue to frustrate clinicians. The diagnosis of neurodegenerative diseases is a superb challenge. Current diagnostic tools for those presenting with suspected AD must measure variation in memory, orientation, judgment, or problem solving, community affairs, participation in home and hobbies, and personal care, and these factors vary by individual and stage of disease progression [4]. Symptoms of PD also vary widely in motor functioning [5]. Evidence of particular importance to ALS pathogenesis includes axonal cytoskeletal disorganization, motor neuron degeneration in the brainstem or spinal cord, weakness or atrophy of voluntary skeletal muscles, microglia activation and subsequent inflammation, and finally amyloid-like fibrils development [6,7]. The high degree of diversity in symptoms presented by patients makes diagnosis challenging between neurodegenerative diseases and within a particular stage of one neurodegenerative disease. However, integrating non-invasive methods for assessing exposures to toxins [3], e.g., extracting DNA and analyzing epigenetic markers, could enhance the diagnostic toolbox for neurodegenerative diseases.

The epigenome consists of processes that modulate the expression of genes external to the gene sequence itself [8]. In each cell, these epigenetic markers may organize the nuclear structure of chromosomes, manage transcription factors’ ability to access DNA, or maintain transcriptional activities performed in the past. Epigenetic markers, including DNA hyper- or hypomethylation and histone modifications such as acetylation, have been found to link environmental exposure to disease outcomes [9]. While CpG sites in the promoter regions of protein-coding genes are usually unmethylated, the hypermethylation of these cytosines could lead to gene silencing and chromosomal instability [10]. Similarly, the acetylation of histone tails typically facilitates the binding of transcription factors needed to attract RNA polymerase for transcription, but the hyperacetylation of histones could cause cell growth arrest, differentiation, and apoptosis [11]. While recent work has linked epigenetic modifications with microglia-induced inflammation in neurodegeneration generally [12], environmental drivers of these changes are not widely studied. Moreover, tissues isolated for epigenetic analysis require invasive biopsy, e.g., of the brain and blood. Identifying biomarkers associated with neurodegenerative diseases in urine would strengthen diagnostic capabilities by introducing a method relying on a non-invasive biospecimen.

Meanwhile, the medical community lacks tools for detecting major neurodegenerative diseases such as Alzheimer’s Disease (AD), Parkinson’s Disease (PD), and amyotrophic lateral sclerosis (ALS) earlier, ideally for signs of disease development prior to onset to slow down disease progression [13]. Patients diagnosed earlier in disease progression could potentially receive treatment in more opportune stages of the disease, when a positive impact on patient quality of life and lifespan is still possible. However, current means of diagnosis of neurodegenerative diseases generally require progressed pathological symptoms to present in a patient, which on average delays diagnosis from the true age of onset by 18 months for AD [14], 6 months for PD [15], and 3 years for ALS [16]. The detection of novel disease-specific biomarkers, such as epigenetic changes, may help in early diagnosis and preventing disease.

Alzheimer’s Disease and related dementias are estimated to nearly double in incidence by 2050 [17]. In 2011, AD incidence was estimated in those aged 65 to 74 years old as 4.0 in every 1000 person-years, 32 of every 1000 person-years for those aged 75 to 84, and 76 of every 1000 person-years for those aged 85 and older [17]. Since 2015, approximately 80% of patients will live one year, 55% of patients will live three years, and only 40% of patients will live longer than five years post AD diagnosis [18]. According to the Parkinson’s Foundation, approximately 18 in every 100,000 person-years are diagnosed with PD each year. While the average survival for someone diagnosed with PD at 9.1 years post diagnosis [19] is longer than that of AD, a recent analysis of the Global Burden of Disease reports PD contributing to the fastest growing levels of disability and prevalence compared to other neurological disorders [20]. ALS incidence within the United States is approximately 2.0 in every 100,000 person-years [21] and increasing by approximately 3% annually [22]. While the incidence for ALS is relatively low compared to other neurodegenerative diseases such as AD or PD, this disease claims 75% of lives within the first five years post diagnosis [23], and only two FDA-approved pharmaceuticals, riluzole and edaravone, are currently able to marginally increase survival and maintain muscle strength, and only if treated early upon disease onset [24]. These facts make a powerful argument for promoting the discovery of novel biomarkers ideally detectable non-invasively by liquid biopsy (e.g., in saliva and urine) to more successfully and reliably predict the risk and progression of disease [13]. However, non-invasive tests are currently unavailable for diagnosing sporadic cases of neurodegenerative disease [25].

In addition to the selection of media for screening, considering geography as a variable allows researchers to associate risk factors such as healthcare access and socioeconomic status with disease susceptibility. For instance, a recent study comparing Medicare records in metropolitan and rural communities found that the incidence of AD was significantly higher among those living in rural communities, with a lower survival rate compared to that of urban communities [26]. It therefore may be the case that geospatial clusters with higher prevalence rates of neurodegenerative diseases within populations can be identified across the United States.

This review aimed to elucidate the environmental factors of neurodegenerative diseases and associated epigenetic biomarkers, specifically for AD, PD, and ALS.

## 2. Materials and Methods

A systematic literature review was conducted, and the subsequent geospatial data were analyzed to determine geographic trends in environmental factors and epigenetic mark studies of neurodegenerative diseases.

### 2.1. Literature Sources

This systematic literature review was planned, organized, and conducted using the Preferred Reporting Items for Systematic Reviews and Meta-Analyses (PRISMA) guidelines. All studies available in the Scopus database prior to December 2021 were screened for eligibility (Supplemental Figures S1 and S2) by a minimum of two researchers to reduce the potential of bias when selecting studies Spot checks by three researchers provided an additional method to confirm adherence to selection criteria prior to analysis.

### 2.2. PRISMA Criteria

Factors or comorbidities associated with epigenetics changes in AD, PD, or ALS cases were searched using keywords “epigen* AND (als OR (amyotrophic AND lateral AND sclerosis) OR (motor AND neuron AND disease) OR (lou AND gehrig’s) OR parkinson’s OR parkinsons OR parkinsonism OR alzheimer’s OR alzheimers) AND (comorbidity or ((toxi* OR contaminant* OR poison*) AND ({exogenous} OR {external} OR {exposure})))”. Papers discussing factors or comorbidities associated with epigenetic changes in AD, PD, or ALS were included, while studies missing either environmental factors, epigenetic changes, e.g., DNA methylation or histone modification, or associated neurodegenerative diseases were excluded from analysis.

Epigenetic markers associated with neurodegenerative diseases were searched using the keywords “epigen* AND (ALS OR (amyotrophic lateral sclerosis) OR (motor neuron disease) OR (Lou Gehrig’s) OR Parkinson’s OR Parkinsons OR Parkinsonism OR Alzheimer’s OR Alzheimers OR neurodegenerative) AND cell AND biomarker”. Studies highlighting cell types or biomarkers, e.g., methylation or histone modification of neurodegenerative diseases were included, while papers for other disorders such as depression, autism, or colorectal cancer were excluded.

### 2.3. Meta Analysis

Additionally, a meta-analysis was conducted from recent literature reviews of environmental factors associated with AD [27], PD [28], and ALS [22]. Environmental factors shared between these three neurodegenerative diseases were identified. Industrial sources of neurodegenerative disease-associated environmental factors were provided by the Environmental Protection Agency (EPA) Chemical Data Reporting for Industrial Processing and Use [29].

### 2.4. Geographic Analysis

Geographic data were visualized for analysis using Geographic Information System ArcGIS Pro 3.0 software. Geographically linked environmental factor exposure studies in human subjects were mapped. EPA-regulated sources of environmental factors shared between AD, PD, and ALS were mapped by industrial location. Finally, US prevalence rates reported to the Centers for Disease Control and Prevention (CDC) were mapped by state or region.

### 2.5. Statistical Analysis

The potential association between industrial toxin locations and neurodegenerative disease prevalence rates was assessed using the likelihood ratio chi-square test. A 95% confidence interval was used to determine significance for the reduction in potential false positives.

## 3. Results

### 3.1. Summary of the Systematic Literature Review

This systematic literature review of environmental factors associated with epigenetic markers in AD, PD, or ALS patients and epigenetic markers associated with neurodegeneration categorically was conducted in Scopus between December 2021 and January 2022 (Supplemental Figures S1–S3). For epigenetic markers associated with neurodegenerative diseases, 523 papers were collected but only 33 studies were included for analysis. For factors or comorbidities associated with epigenetics changes in AD, PD, or ALS cases, 464 papers were identified but only 36 studies were included in this review. 

### 3.2. Environmental Toxins Common to AD, PD, and ALS

**Figure 1:**
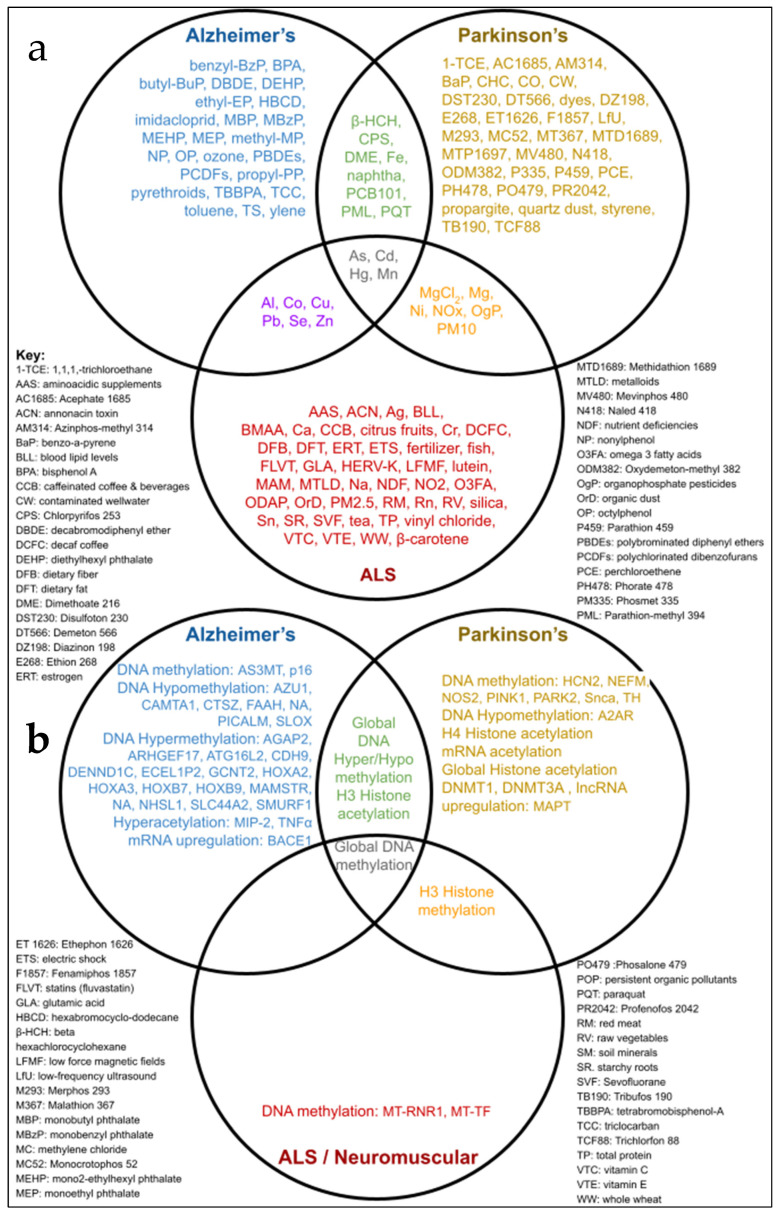
Venn diagram identifying environmental factors and epigenetic changes common to Alzheimer’s Disease, Parkinson’s Disease, and amyotrophic lateral sclerosis (ALS). Venn diagrams show the associations between AD, PD, and ALS with (**a**) environmental toxins and (**b**) epigenetic changes. Factors only associated with one neurodegenerative disease are shown in each outside circle (blue, red, or yellow text), whereas toxins shared by only two neurodegenerative diseases are shown in segments within two neurodegenerative disease circles (purple, orange, or green text). Toxins implicated in Alzheimer’s Disease, Parkinson’s Disease, and amyotrophic lateral sclerosis are shown in the central shared segment (gray text).

A total of 127 factors were supported by the literature to be associated or putatively associated with at least one of the three neurodegenerative diseases studied. For AD, 55 environmental factors were identified [27], for PD a total of 51 toxins with high odds ratios was found [28], and for ALS, 74 environmental toxins were computed [22]. Only four chemicals were shared across these three major neurodegenerative diseases, all representing elements belonging to the group of toxic metals: mercury (Hg), manganese (Mn), cadmium (Cd), and arsenic (As) (Figure 1).

**Figure 2:**
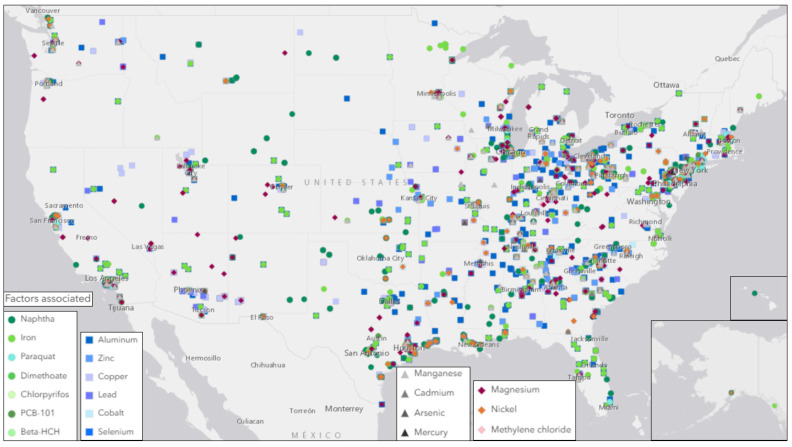
At-risk populations by industrial source locations of environmental factors associated with Alzheimer’s Disease, Parkinson’s Disease, and amyotrophic lateral sclerosis. Toxins associated with Alzheimer’s Disease (AD), Parkinson’s Disease (PD), and amyotrophic lateral sclerosis (ALS) are mapped geographically in ArcGIS. Toxins associated with all three neurodegenerative diseases are shown by gray triangles. Toxins associated with PD and ALS are shown by pink diamonds, AD and PD are green circles, and AD and ALS are shown with blue squares. Data on locations of toxin sources were taken from EPA’s Chemical Data Reporting for Industrial Processing and Use Report [28].

Figure 2 maps the source locations regulated by the EPA for the industrial manufacturing and distribution of toxicants also associated with AD, PD, or ALS [29]. Sources of heavy metals in the U.S. were primarily located in Eastern states, and included primary metal manufacturing, fabricated metal product manufacturing, petrochemical manufacturing, plastics material and resin manufacturing, wood product manufacturing, pharmaceutical and medicine manufacturing, and electrical equipment, appliance, and component manufacturing. Toxin sources tended to be more dispersed and less concentrated in large clusters within Western regions.

**Figure 3:**
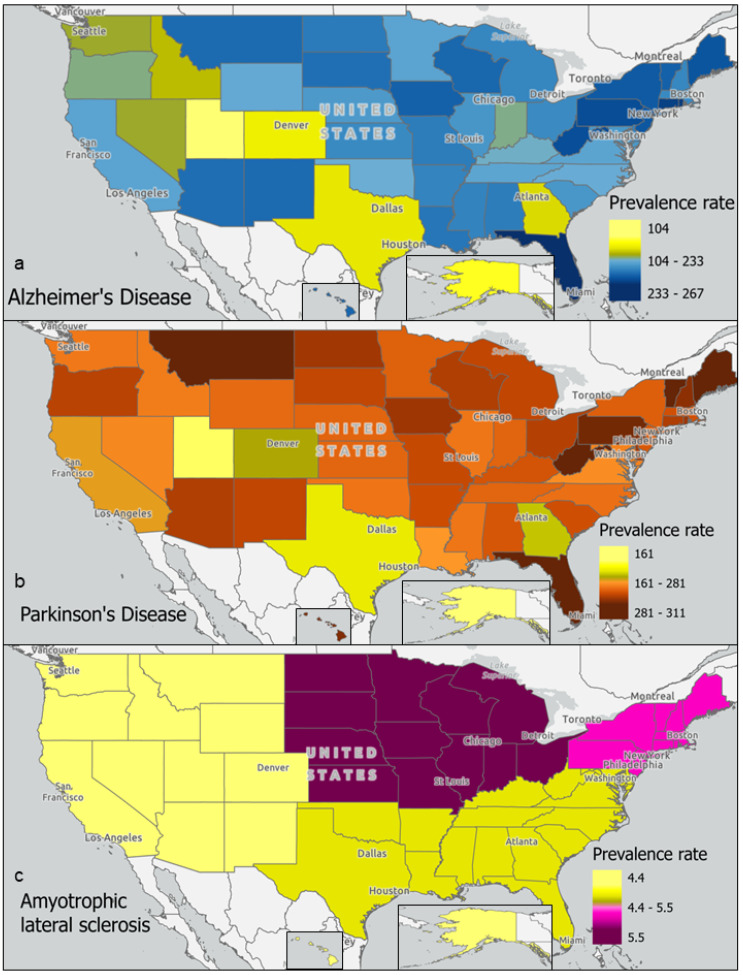
Neurodegenerative disease prevalence rates in the United States. Prevalence rates (per 100,000 persons) of (**a**) Alzheimer’s Disease, (**b**), Parkinson’s Disease, and (**c**) amyotrophic lateral sclerosis are mapped geographically in ArcGIS. Prevalence rates are reported per 100,000 persons.

Meanwhile, prevalence rates of each neurodegenerative disease provide potential study locations for markers of disease. This paper used AD prevalence data reported for 2020 [17] to adjust for population density with 2020 U.S. Census Bureau national and state population estimates. The prevalence of PD for 2016 reported by the Parkinson’s Foundation [30] was further adjusted for population counts in 2016 using U.S. Census Bureau national and state population estimates. These rates for AD and PD, as well as the rates of ALS prevalence reported to the CDC, were mapped by region in Figure 3 [31]. Prevalence rates of AD reported per 100,000 people (Supplemental Table S1) were above the mean (184) in states including Florida (267), Rhode Island (227), Connecticut (225), and Pennsylvania (219). Rates for PD were above the mean (247) in states including Florida (310), Maine (300), Vermont (288), and Montana (288). Rates for ALS were above the mean (4.9) in midwestern (5.5) and northeastern (5.1) regions (Figure 3). Generally, neurodegenerative disease prevalence rates tend to be lower in Western states compared to Northeastern states. Nevertheless, elevated prevalence rates per 100,000 people in states such as Florida, New Mexico, Montana, and Arizona suggest a need to study these regions for AD (266.9, 204.1, 203.6, and 202.1, respectively) and PD (310.5, 254.7, 287.8, and 259.7, respectively).

Furthermore, statistical analyses showed regional neurodegenerative disease prevalence rates to be significantly correlated with the locations of industrial sources of toxins linked to neurodegenerative diseases (Supplemental Table S2). The four toxic metals (i.e., arsenic, cadmium, manganese, and mercury) were significantly associated (*p* = 0.0138) with states with high AD, PD, or ALS prevalence rates. Additionally, these toxic metals, as well as others (i.e., iron, aluminum, cobalt, copper, lead, zinc, magnesium, and nickel), were joined by other environmental toxins (i.e., beta-HCH, chlorpyrifos, dimethoate, naphtha, paraquat, PCB-101, methylene chloride, and selenium) when correlating to a combined neurodegenerative disease prevalence rate (*p* < 0.0001), merging all AD, PD, and ALS prevalence rates.

### 3.3. Environmental Factors Associated with Epigenetic Markers in AD, PD, and ALS

Few studies have reported associations between exposure to environmental toxins and epigenetic markers in patients with neurodegenerative diseases. The largest studies by cohort size have been conducted in the United States (Supplemental Figure S4). Toxins geographically linked with AD, PD, or ALS prevalence rates include welding fumes from blood samples collected from participants in the Midwest US [32], air pollution from brain matter collected in Mexico [33], and from blood in Italy and China [34], arsenic presence in maternal peripheral blood and neonatal cord blood in Argentina and Bangladesh [35], and organophosphate presence in blood samples collected in Californian counties [36] (Supplemental Figure S4).

**Figure 4:**
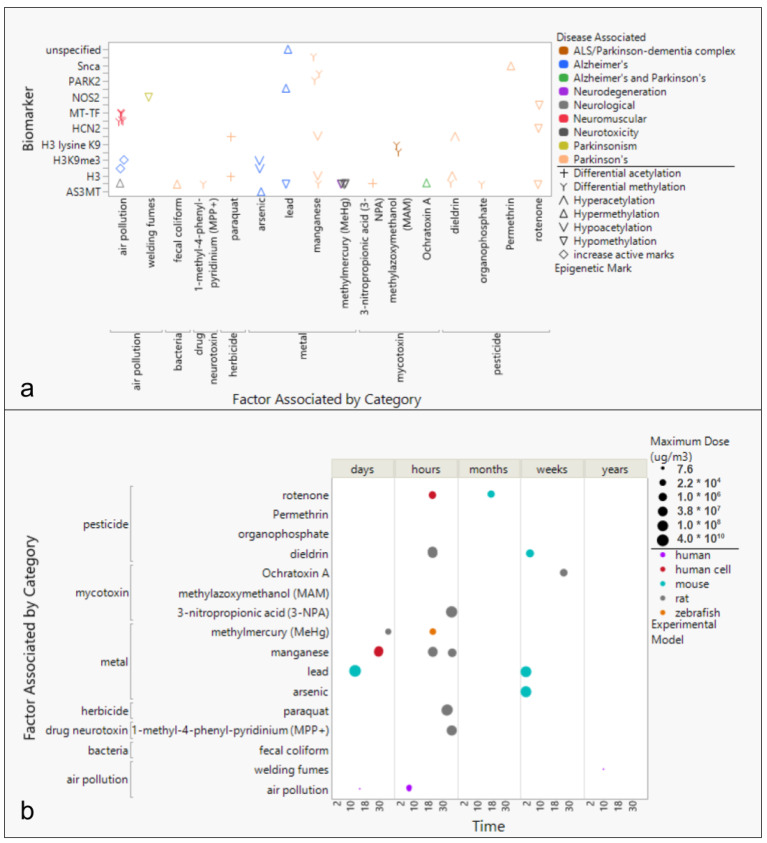
Trends of epigenetic biomarkers associated with exposure to environmental factors by time and dose for Alzheimer’s Disease, Parkinson’s Disease, and amyotrophic lateral sclerosis. (**a**) Studies of exposure to environmental toxins reported changes in epigenetic biomarker pattern (acetylation or active marker changes) outcomes that are known to be associated with neurodegenerative diseases. (**b**) Experimental models and human cohort or case–control studies of AD, PD, and ALS reported changes to epigenetic markers based on time and dose response.

Analysis of epigenetic markers and exposure to environmental toxins in neurodegenerative disease cases have revealed associations between toxins, methylation or acetylation, and disease. AD was associated with air pollution (histone methylation) [33] and lead (Pb, hypermethylation) in the p16 promoter [10], whereas PD has been associated with paraquat (differential acetylation) [37], dieldrin (hyperacetylation) [11], and manganese (hypoacetylation) [38] (Figure 4).

Studies of the effects of toxic exposures have primarily been performed with animal models (Figure 4). Mouse experimental models have generally been studied for longer time scales, e.g., from days to months, compared to rat or human cell models, e.g., typically hours to weeks (Figure 4). Investigations of rat and mouse models studied higher exposure doses compared to other experimental models. Unlike other toxins, neurodegeneration effects from air pollution exposure have been exclusively studied in human populations [33]. Mycotoxins and cyanotoxins have exclusively been studied in rats [39,40,41]. Mouse models have primarily been used to study pesticides, e.g., dieldrin and rotenone [11,42], as well as toxic metal exposures, e.g., arsenic and lead [43,44].

### 3.4. Epigenetic Markers of Neurodegenerative Diseases

**Figure 5:**
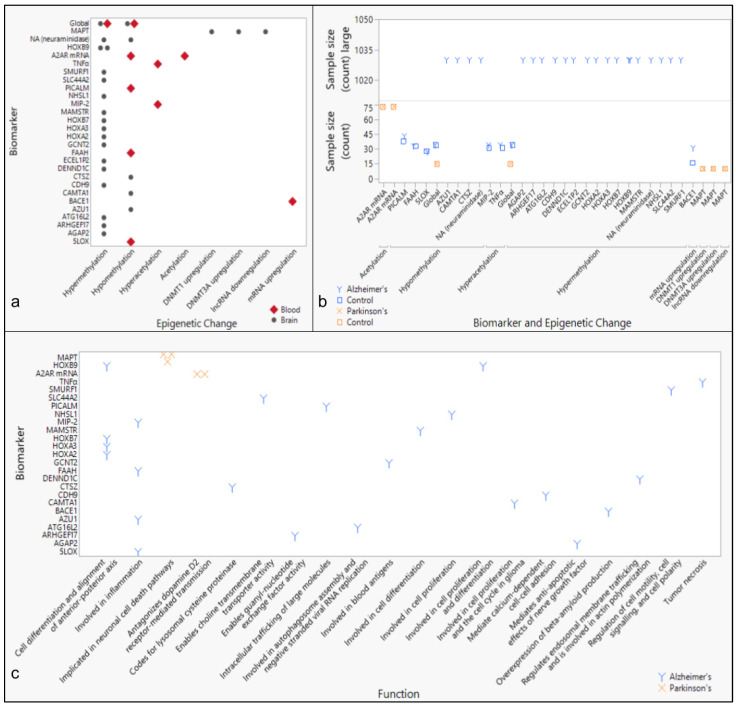
Epigenetic biomarkers by tissue type and associated pathways in Alzheimer’s and Parkinson’s Disease. (**a**) Biomarkers identified in the literature are grouped by epigenetic changes and tissue type. (**b**) Subject counts for patient or control specimens are plotted by biomarkers categorized by epigenetic changes associated with disease. (**c**) Biomarkers are further grouped by primary general or disease-specific function(s) and associated neurodegenerative disease.

Most studies report global hypermethylation or hypomethylation as a biomarker of AD and PD (Figure 5). While the majority of the hypermethylation signatures were found in brain tissue, hypomethylation signatures were identified in both brain and blood samples. Within each study, the subject counts averaged approximately 30 patients and 30 controls, except for one study [45], in which 1030 specimens were collected from patients with AD (Figure 5). Furthermore, most biomarkers studied in AD patient specimens were associated with structural changes and inflammation, whereas biomarkers associated with PD were linked to cell cycle regulation or metabolic processes (Figure 5). Overall, differential global DNA methylation was implicated in studies associated with AD, PD, and ALS (Figure 1).

## 4. Discussion

The purpose of this review was to identify environmental factors and subsequent epigenetic changes associated with neurodegenerative diseases. Previous reports have shown epigenetic changes to be driven by environmental exposures, and neurodegeneration generally to be associated with exposure to heavy metals. This paper specifically identifies an association between three neurodegenerative diseases (AD, PD, and ALS) and the toxic metals As, Cd, Mn, and Hg (Figure 1). In addition, geographic locations of industrial sources of toxins associated with neurodegenerative diseases, including heavy metals, were also found to be significantly associated with neurodegenerative disease prevalence rates by state (Figure 2 and Figure 3, Supplemental Table S2). Meanwhile, epigenetic changes such as differential methylation of histones and DNA have been associated with exposure to air pollution and lead in AD, while differential acetylation has been associated with exposure to paraquat, dieldrin, and manganese in PD. (Figure 4). These epigenetic changes are further found to be associated with structural changes and inflammation in AD patients and cell cycle regulation or metabolic processes in PD patients (Figure 5).

### 4.1. Neurodegenerative Disease Prevalence and Exposure to Environmental Toxins

Metals and pesticides have been linked to the pathogenesis of neurological disorders (Figure 1; Supplemental Table S3) [46]. Long-term exposure to neurotoxins, occupationally or from residential proximity to manufacturing and distribution sites of toxins associated with AD, PD, and ALS (Figure 2), is hypothesized to contribute to an increase in prevalence rates of neurodegenerative diseases regionally from the present review. AD and PD prevalence rates and toxin exposure align geographically, except for Montana. Furthermore, ALS-associated toxin manufacturing and prevalence rates align to AD- and PD-associated toxin sources and prevalence rate trends with higher rates in the Northeastern states and lower rates in the western states. However, the aggregated ALS prevalence rates reported by region provide data that are too broad for thorough analysis. Since Florida is in the southern region and Washington is in the western region, they are reported to have lower than average (4.9) prevalence rates of 4.68 and 4.41 cases per 100,000 people, respectively [31]. However, studies reporting incidence rates of ALS cases in these states have reported higher than average (1.0) incidence rates of 1.73 per 100,000 person-years in Florida and 1.84 per 100,000 person-years in Washington [21]. This discrepancy between aggregated prevalence rates and case incidence rates may potentially be explained by localized factors, such as an unusual age demography, producing outlier states within a given region. Therefore, we expect that prevalence rates calculated with higher resolution in more localized regions that are also age-adjusted would offer more accurate estimates of susceptible populations.

Additionally, our analysis was limited to comparing the presence of toxicants in sites regulated by the EPA. A future investigation pooling these data with other source data with potential sites unregulated by the EPA may reveal the presence of toxicants in more locations. Furthermore, another study could compile toxicant levels for potential dose-level analysis. This review’s analysis of the mere presence of toxicants in regulated sites could be informed by the addition of data including concentration of toxicants in more source locations. It is important to consider that human exposure to toxicants is likely in more sources than manufacturing and distribution locations. After all, environmental factors associated with lifestyle or residential exposure include diet (e.g., citrus fruit, dyes, dietary fat, dietary fiber, fish, red meat, raw vegetables, and starchy roots) or indicators of diet (e.g., omega 3 fatty and glutamic acids) (Figure 1).

Furthermore, it is of note that metalloids and metals (e.g., As, Cd, Hg, and Mn) have known human neurotoxic effects ranging beyond the three neurodegenerative diseases highlighted in this review. Arsenic toxicity is known to cause peripheral neuropathy, particularly a sensory–motor axonopathy [47]. Long-term exposure to cadmium has been shown to lead to neuronal cell apoptosis, impaired neurogenesis, and altered gene expression and epigenetic effects from accumulation in the brain [48]. The neurobehavioral and neurochemical indications of neurodevelopmental disease from exposure to any level and form of mercury have been well documented [49]. Manganism, a neurodegenerative disorder caused by an accumulation of manganese in the brain, shares symptoms with PD patients—particularly dysfunction in the basal ganglia [50]. Neurotoxic effects on the body separate from the formal diagnoses of AD, PD, and ALS need to be acknowledged and considered when associating metalloids and metals with specific neurodegenerative diseases.

### 4.2. Environmental Toxins Common to Neurodegenerative Diseases and Associated Epigenetic Changes

Our study shows that epigenetic changes from exposure to air particulate matter, metals, and pesticides are the most studied neurodegenerative disease factors examined by geographic location (Supplemental Figure S4). However, we report very few geographic studies relevant to human disease, due to the high number of studies using experimental models, particularly mice, rats, or cell cultures grown ex situ (Figure 4). Studies were primarily located in regions of the U.S., Europe, and East Asia when geospatial data were shared (Supplemental Figure S4). While this sampling choice may be due to institutional convenience, this limited geographic analysis in this review. Additionally, the high number of studies choosing to use animal models over human-based models also places some doubt on the ability to translate results to human subjects. While cell cultures are not representative of the complex ecosystem within the human body, the choice to conduct exposure studies with human cells and tissues in cultures or 3D tissue cultures on chips could offer a preferred alternative when later translating results to human systems. Meanwhile, the choice to utilize non-human primate models may offer a means to study interactions more translatable to humans but necessitates prior study with other models. We can also note that computational modeling may offer yet another alternative, particularly as technological advances in machine learning could make predictive modeling more accurate. While often ethically necessary, the use of short-lived animal models to determine epigenetic changes from the exposure to toxins limits the certainty of the data analyzed herein.

#### 4.2.1. Epigenetic Changes Associated with Air Pollution

Exposure to air pollution levels above the US EPA and WHO PM10/PM2.5 standards [51] was associated with histone post-translational modifications (HPTMs). The loss of the HPTMs H3K9me2 and H3K9me3 needed to repress lineage-specific tissue and developmental-coding genes has been observed in patients with AD [37]. While no studies have specifically associated exposure to environmental stressors with DNA methylation in ALS, the methylation of mtDNA transfer RNA phenylalanine (MT-TF) and 12S ribosomal RNA (MT-RNR1) genes has been associated with occupational PM1 air pollution exposure in neuromuscular disease [34]. The mechanistic components of epigenetic changes driven by exposure to air particulate matter pollution have been suggested to include parental allele imprinting, histone withholding, noncoding RNAs, and mtDNA [52]. However, researchers have acknowledged the need for dose and time effects, genetic make-up, immune susceptibility, activity patterns, socio-economic status, and lifestyle factors such as nutrition to be considered when fully characterizing the mechanisms of epigenetic changes in individuals.

#### 4.2.2. Epigenetic Changes Associated with Toxic Metals

In patients with AD exposed to lead, hypermethylation of the tumor suppressor gene p16 was observed in blood [10]. Globally, however, lead exposure associated with AD has driven a global hypomethylation of genes associated with neurodevelopment or cognitive function in neural cells [53]. Manganese exposure in PD models resulted in histone H3 and H4 hypoacetylation [38]. Exposure to manganese has also been shown to differentially methylate 529 targeted genes [54], and PINK1, PARK2, and TH specifically, which are genes assisting with neuronal development and function, dopamine metabolism, and proteolysis biological pathways, the dysfunction of which has been associated with PD [55]. Targeted gene analysis of arsenic exposure has revealed global hyperacetylation in hippocampal tissue [43], a brain region known to be essential for learning and memory. Furthermore, hypermethylation of AS3MT has been observed in the blood of patients with AD and arsenic exposure in the gene coding for the primary methyltransferase needed for arsenic metabolism [35]. More generally, neurodegeneration in stem cells [56] and neurotoxicity in the cerebrum, regulating movement and temperature [57], from methylmercury exposure has driven global hypomethylation in animal models. Also associated with PD, blood analysis of welding fume exposure has been observed to drive NOS2 hypomethylation, known to increase the production of inducible nitric oxide synthase (iNOS) and thereby the pro-inflammation mediator nitric oxide [32]. As for the mechanisms of action, exposure to lead has been associated with hypomethylation and histone H3 and H4 modifications (H3K9ac, H4K8/K12ac, H3K4me2), exposure to arsenic has been shown to drive hypo- and hypermethylation, as well as histone H3 and H4 modifications (H3S10p, H2AXp, H3K9/K27/K16/K18ac, H3K4me2/me3, H3K9me2, H3K27me3, H3R3me2, H3R17me2), and exposure to mercury has been shown to drive hypo- and hypermethylation and histone H3 modifications (H3ac, H3K27me3) [58]. Oxidative stress and disruption of the cell metabolism have specifically been implicated as mechanistic drivers of epigenetic alterations upon exposure to toxic metals.

#### 4.2.3. Epigenetic Changes Associated with Pesticides and Herbicides

Differential acetylation of histone H3 has been observed in PD models exposed to paraquat, an herbicide [37]. Exposure to permethrin, a pyrethroid insecticide, in a Wistar rat PD model drove hypermethylation of SNCA, contributing to the downregulation of the dopamine-synthesis pathway, in the striatum, a region of the brain responsible for movement and motivation [59]. Exposure to dieldrin, an organochlorine pesticide, has been shown to lead to differentially methylated Nr4a2 and Lmx1b genes, responsible for dopaminergic neuron development and maintenance [60], and hyperacetylation of histones H3 and H4 [11] in mouse neuronal cells. In terms of hypomethylation, exposure to rotenone, a naturally occurring hetero-pentacyclic pesticide, was shown to drive hypomethylation of HCN2, responsible for regulating neuronal plasticity, and NEFM, driving axonal growth and transport, in kidney cell cultures associated with PD [61], and global hypomethylation in liver cells [42]. Furthermore, in blood and saliva samples of individuals with PD, global methylation analysis revealed differential methylation in those exposed to organophosphates, synthetic compounds containing phosphorus [36]. Organophosphates are often applied as pesticides, herbicides, and flame retardants but are toxic to humans in high doses or after long-term exposure to low doses by overloading acetylcholine in cholinergic synapses, leading to oxidative stress and neuroinflammation [62]. Meanwhile, exposure to paraquat has been shown to induce histone H3 acetylation, resulting in a decreased total histone deacetylase activity [37]. Exposure to dieldrin similarly induces H3 and H4 acetylation and has been shown to result in histone acetyltransferase accumulation. Ultimately, histone modification has been determined to be a key epigenetic change upon exposure to paraquat and dieldrin in a dose- and time-dependent manner [63].

#### 4.2.4. Epigenetic Changes Associated with Mycotoxins and Cyanotoxins

Exposure to Ochratoxin A, a naturally occurring organic compound leading to neuronal oxidative damage associated with both AD and PD, drove global hypermethylation in rat kidney cells [39]. Nevertheless, the potential role of Ochratoxin A in the etiology of specific neurodegenerative diseases should not be overstated, as it is a highly ubiquitous toxin in agricultural products. Meanwhile, prenatal exposure to methylazoxymethanol (MAM), a developmental neurotoxin, has been associated with postnatal methylation changes in histone H3 in rat models of schizophrenia [41]. The effects of Ochratoxin A on the human body have been associated with mechanisms including DNA adduct formation, protein synthesis inhibition, cellular energy production alteration, oxidative stress, apoptosis, mitosis changes, cell cycle arrest, and cytokine pathway interference [64]. As for MAM, a model with schizophrenia was studied to determine epigenetic changes around the Drd7 gene and showed differential DNA methylation in the co-factors DISC1, Syp, and Dtnbp1 [65]. Meanwhile, deoxynivalenol (DON) was not a subject of the studies drawn on in this review, and yet this mycotoxin is significantly important to both human and farm animals potentially exposed through food sources. The fast but species-specific absorption rates present a threat because epigenetic modifications have been found to be induced in porcine oocytes [66]. Recent research also suggested the potential for DON to cross the blood–brain barrier, a major concern for the potential to drive neurodegeneration, when low concentrations of DON were detected in the brains of mice, chickens, and fish [67]. Further study into the possibility of DON to drive epigenetic changes would help to inform its role in neurodegeneration.

Mycotoxins and cyanotoxins have been associated with ALS and PD since the Western Pacific Amyotrophic Lateral Sclerosis and Parkinsonism–Dementia Complex (ALS-PDC) was first studied in the Chamorro people of Guam [68]. The cyanotoxin β-N-methylamino-L-alanine (BMAA) was originally implicated for its neurotoxin effects when dietary changes in the native population occurred simultaneously with a decline in the incidence of ALS-PDC in the region. MAM was included as another component of the cycad seed, an aglycone of cycasin that induces DNA lesions promoting transcriptional mutagenesis from the production of faulty RNAs [69]. Furthermore, mistakes in messenger RNAs have been shown to generate mutant proteins, including amyloid-like proteins associated with human aging [70]. As for mycotoxins, another ALS cluster discovered in France linked the volatile mycotoxin gyromitrin to the disease due to its potential to produce free radicals that damage DNA upon consumption [71]. Therefore, there is significant historical reasoning to focus epigenetics research on mycotoxins and cyanotoxins associated with neurodegenerative diseases.

### 4.3. Differential DNA Methylation in Neurodegenerative Diseases

Studies assessing methylation patterns represented 86% of the studies reporting epigenetic changes associated with neurodegenerative diseases (Figure 5; Supplemental Table S4). Global methylation analysis in AD patients revealed that 11,822 CpGs were hypermethylated CpGs and 6073 CpGs were hypomethylated in brain tissue [72]. In PD samples, 31 differentially methylated regions were identified [73]. Thirteen regions containing CpG sites were hypermethylated and eighteen regions were hypomethylated in whole blood [73]. Genome-wide DNA methylation in post-mortem brains of sporadic ALS patients has revealed 38 differentially methylated sites when compared to controls [74]. In DNA extracted from blood, genome-wide analysis has revealed differential methylation associated with age of onset at three CpG sites [75]. Global hypermethylation (mean mC% above the top whisker of the control) was observed in spinal cord tissue, but not in whole blood samples of individuals diagnosed with ALS [76]. Targeted gene analysis or methylation signatures have also been researched for these neurodegenerative diseases. Patients with AD were observed to exhibit hypomethylation of the PICALM gene in whole blood [77], of the Alox5 promoter in peripheral blood mononuclear cells (PBMCs) in late-onset cases [78], and of FAAH in PBMCs [79] when compared to controls. In PD cases, hypermethylation has been observed for the MAPT promoter in brain tissues [80], and hypomethylation of the A2A receptor gene in PBMCs of sporadic cases [81]. In Europe and the US, 40% of familial ALS cases and 8% of sporadic ALS cases have been attributed to C9orf72 mutation, with higher prevalence in Scandinavian ancestry [82,83]. Specifically, hypermethylation of the C9orf72 expansion CpG island has been observed in blood of ALS patients [84], largely using quantitative real-time polymerase chain reaction (qPCR) techniques. Nevertheless, many biomarkers associated with neurodegenerative diseases are more commonly seen in brain tissue as opposed to blood. This may make the detection of epigenetic patterns through wastewater-based epidemiology difficult, since brain tissue is not likely to be found in influent wastewater. Further discussion is shared in the Supplementary Material.

Ultimately, monitoring environmental factors and the resulting epigenetic changes most associated with disease would reduce the delay in diagnosis for neurodegenerative diseases such as AD, PD, and ALS. Further research into the epigenetic effects (e.g., differential acetylation or methylation) from exposure to the four toxic metals common to AD, PD, and ALS (e.g., arsenic, cadmium, mercury, and manganese) would reveal their sensitivity and specificity as neurodegenerative disease biomarkers. Given enough research, a library could be developed to curate and evaluate these biomarkers to help prioritize their use in future diagnostic screening, as has been developed for aging [85] and (as recommended) for cancer [86] biomarkers. Integrating non-invasive screening methods such as monitoring environmental factor exposure and epigenome status with currently available clinical tools including MRI, PET scans, and behavioral testing [18] could reveal more specific and early signs of neurodegeneration.

### 4.4. Considering a Lifetime of Exposures

While this review shares the reported effects of exposure from each toxin previously analyzed, it is important to recognize that exposure events are much more complex in a human’s lifetime. Exposures to multiple toxins with varying doses longitudinally in time confound our ability to predict disease onset and progression. Of the 19 studies found to include dose and time effects, only 5 reported using human-related models studying only three toxicants (i.e., air pollution, welding, and rotenone) (Figure 4b). An individual’s exposome must be considered to better understand the risk of neurodegeneration holistically [22]. Furthermore, toxins of emerging concerns not yet studied for their epigenetic effects could pose threats not yet revealed. Micro- and nano-plastics as well as per- and polyfluoroalkyl substances (PFAS) sourced from plastics and pharmaceutical residues are of particular interest in research today, and yet their potential contribution to the etiology of neurodegenerative diseases is not included in the available studies within this review. Studies of exposure to a wide range of toxins, in varying concentrations of doses, and in multiple exposure events over the course of an individual’s lifetime are needed to better understand the realistic impact of resulting epigenetic changes on neurodegeneration.

### 4.5. Review Time Window

The authors would also like to note that funding was made available to support this literature review through the year 2021. Since the preparation of the present manuscript, a number of studies have been published, particularly in regard to epigenetic changes associated with neurodegenerative diseases. Noteworthy ones include a study of patients with late-onset AD showing a higher rate of differential expression of CHI3L2 in males exhibiting depression as a comorbidity [87], a review of epigenetic changes currently associated with age-related disorders highlighting investigations of AD and PD in both human and animal models [88], and a study finding epigenetic changes in a CpG site near the KALRN gene previously associated with AD [89]. We hope other studies are forthcoming that also add additional support for relating environmental factor exposures to epigenetic changes associated with neurodegenerative diseases to the scientific literature.

## 5. Conclusions

Epigenetic changes caused by exposure to heavy metals are currently being studied [90]. Nevertheless, studies of epigenetic effects from exposure to other toxins highly associated with each neurodegenerative disease are minimal. While the cyanotoxin MAM has been studied for its roles in driving epigenetic changes in neurodegenerative diseases, the neurotoxin most associated with ALS to date, BMAA [22], is notably absent from this research. Studying epigenetic changes that occur specifically from environmental factors most associated with each neurodegenerative disease could potentially reveal disease-specific biomarkers facilitating an earlier diagnosis. AD research has focused on air pollution and heavy metals, while PD research has focused on pesticides and the heavy metal manganese (Figure 4).

Higher doses have primarily been tested in animal models compared to human models, which is ethically necessary when incidental exposures are not captured. However, translating results from direct exposure observed in animals to changes in pathogenesis seen in humans can be problematic and potentially unproductive when attempting to inform on the impacts of exposure in the clinical setting. Except for air pollution, whose effects have primarily been studied in human subjects, reported effects of environmental toxin exposure on neurodegeneration may not translate from animal models to humans. Research using rodents or other short-lived species is unable to produce longitudinal studies necessary to mimic long-latency diseases such as neurodegenerative diseases. However, considering non-human primates for research must include ethical and financial bureaucracy. Future screening for methylation changes in individuals will therefore need to be coupled with the validation of environmental toxin exposure events.

Continued study could lead to clinical applications of epigenetic changes and environmental factors as biomarkers. Research into familial variants and other genetic predispositions leading to neurodegeneration (e.g., hexanucleotide repeat expansion mutations in the promoter region of C9orf72, known to drive the ALS pathology; SCA2 and SCA3 expansion mutations, LRRK2 mutations, and single-nucleotide polymorphisms (SNPs) in SNCA, MAPT, and GBA genes possibly driving PD pathogenesis; and SNPs in APOE, widely known to drive AD pathology) assists in understanding mechanisms of disease progression [91], and could be leveraged in assisting diagnosis using scales such as polygenic risk scores (PRS) [92]. Nevertheless, primarily studying genetic mutations associated with disease has been suggested to be antiquated in neurodegenerative etiology research today, as the role of environmental triggers in most cases has been reported [93]. Genomic sequencing may expose a predisposition to disease by identifying genes associated with disease; however, epigenomic profiling more accurately describes gene expression contributing to disease pathogenesis. As gene expression is regulated by epigenetic changes such as DNA methylation, identifying epigenetic biomarkers of disease that could be tracked in a variety of methods would increase the likelihood of detecting disease. Ultimately, surveilling disease-associated environmental factors such as the toxic metals As, Cd, Mn, and Hg, as well as methylation patterns in populations at various geographic locations, may aid in identifying at-risk populations at a spatial level.

## Data Availability

The original contributions presented in this study are included in the article/Supplementary Material. Further inquiries can be directed to the corresponding author.

## Supplementary Materials

The following supporting information can be downloaded at: https://www.mdpi.com/article/10.3390/toxics13020114/s1. 1. PRISMA Diagram of biomarkers in wastewater. Supplemental Figure S1. PRISMA Systematic literature review diagram of epigenetic marks associated with neurodegenerative diseases; 2. PRISMA Diagram of Epigenetic biomarkers in urine and feces. Supplemental Figure S2. PRISMA systematic literature review diagram of factors or comorbidities driving epigenetic marks associated with Alzheimer’s Disease, Parkinson’s Disease, or amyotrophic lateral sclerosis; 3. Publication Trends. Supplemental Figure S3. Publication trends in epigenetics and factors/comorbidities associated with epigenetics in neurodegenerative disease PRISMA searches; 4. Study locations for neurodegenerative disease-associated toxins driving epigenetic. Figure S4. Location and subject count of studies associating exposure of environmental toxins with Alzheimer’s Disease, Parkinson’s Disease, and amyotrophic lateral sclerosis; 5. Prevalence rates. Supplemental Table S1. AD, PD, and ALS Prevalence rates (per 100,000 persons) by US state; Supplemental Table S2. Statistical analysis comparing neurodegenerative disease prevalence rates and toxins associated with Alzheimer’s Disease, Parkinson’s Disease, and/or ALS by state; 6. Additional Outstanding Questions [94,95,96,97,98,99]; 7. Review Sources Metadata. Supplemental Table S3. Factors and Comorbidities Driving Epigenetic Marks Associated with Alzheimer’s Disease, Parkinson’s Disease, and Amyotrophic Lateral Sclerosis. Supplemental Table S4. Epigenetic Marks Associated with Neurodegenerative Diseases.
