# Supplementary material for: Epigenetic Biomarkers Driven by Environmental Toxins Associated with Alzheimer’s Disease, Parkinson’s Disease, and Amyotrophic Lateral Sclerosis in the United States: A Systematic Review
Source: Toxics. 2025 Jan 31;13(2):114. doi: 10.3390/toxics13020114 (PMC11860158; doi:10.3390/toxics13020114)
Supplement: Supplementary file 1 [file toxics-13-00114-s001.zip › toxics-3415312-supplementary.pdf]

Systematic Review

Epigenetic Biomarkers Driven by Environmental Toxins Associated with Alzheimer's Disease, Parkinson's Disease, and Amyotrophic Lateral Sclerosis in the United States: A Systematic Review

Melanie Engstrom Newell [1,2,3], Anumitha Aravindan [1,2,4], Ayesha Babbrah [1,2,4] and Rolf U. Halden [1,2,3,4,5,*,†]

Biodesign Institute, Arizona State University, Tempe, AZ 85287, USA
2 Biodesign Center for Environmental Health Engineering, USA
3 School for Engineering of Matter, Transport and Energy, USA
4 Barrett The Honors College, USA
5 School of Sustainable Engineering and the Built Environment, USA
* Correspondence: rolf.halden@asu.edu
† Current address: Biodesign Institute Building B, 1001 S McAllister Ave, Tempe, AZ 85287, USA.

Supplemental Material

1 PRISMA Diagram of biomarkers in wastewater.

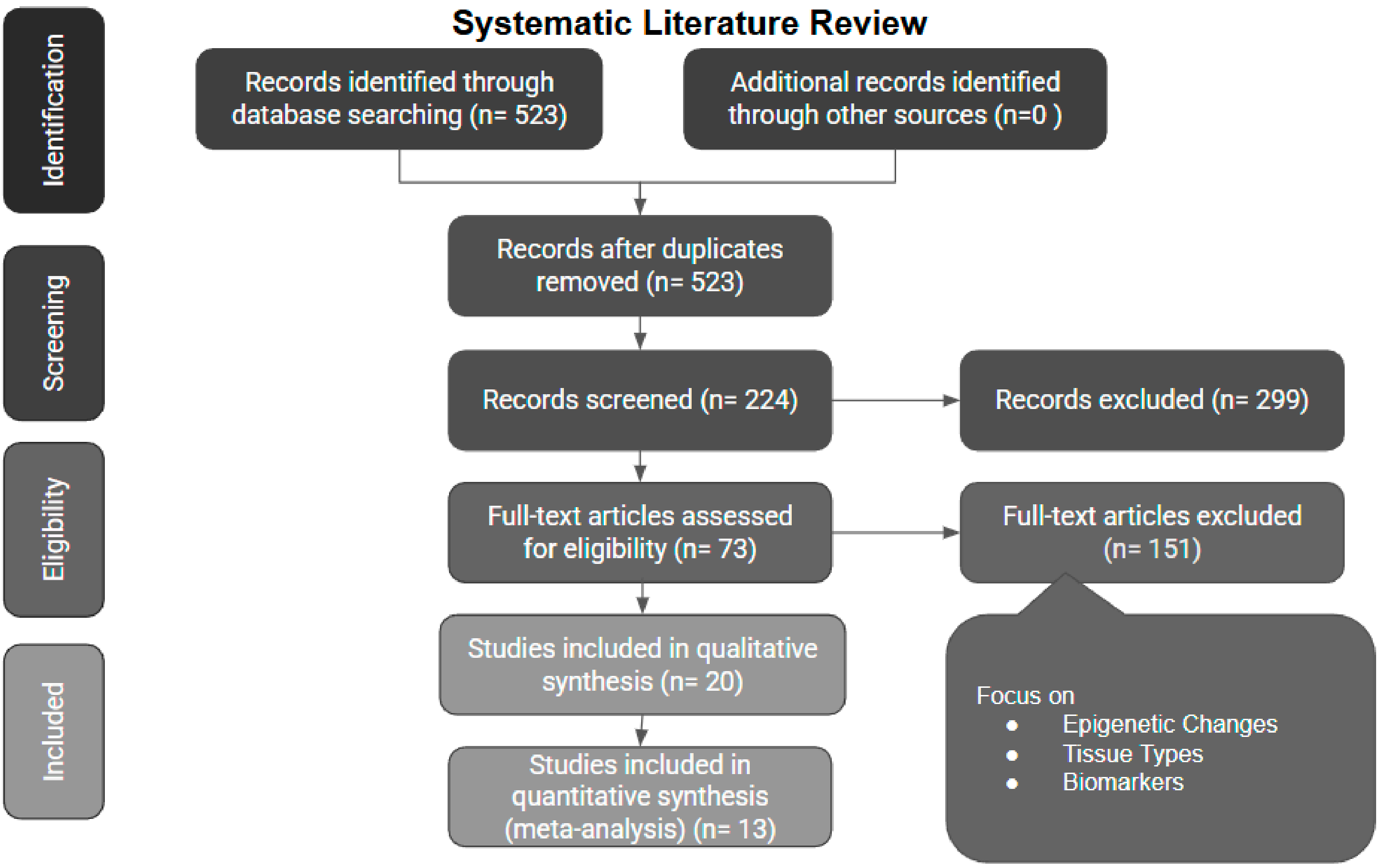

Supplemental Figure S1. PRISMA Systematic literature review diagram of epigenetic marks associated with neurodegenerative diseases. Key words: epigen* AND (ALS OR (amyotrophic lateral sclerosis) OR (motor neuron disease) OR (Lou Gehrig's) OR Parkinson's OR Parkinsons OR Parkinsonism OR Alzheimer's OR Alzheimers OR neurodegenerative) AND cell AND biomarker

2 PRISMA Diagram of Epigenetic biomarkers in urine and feces.

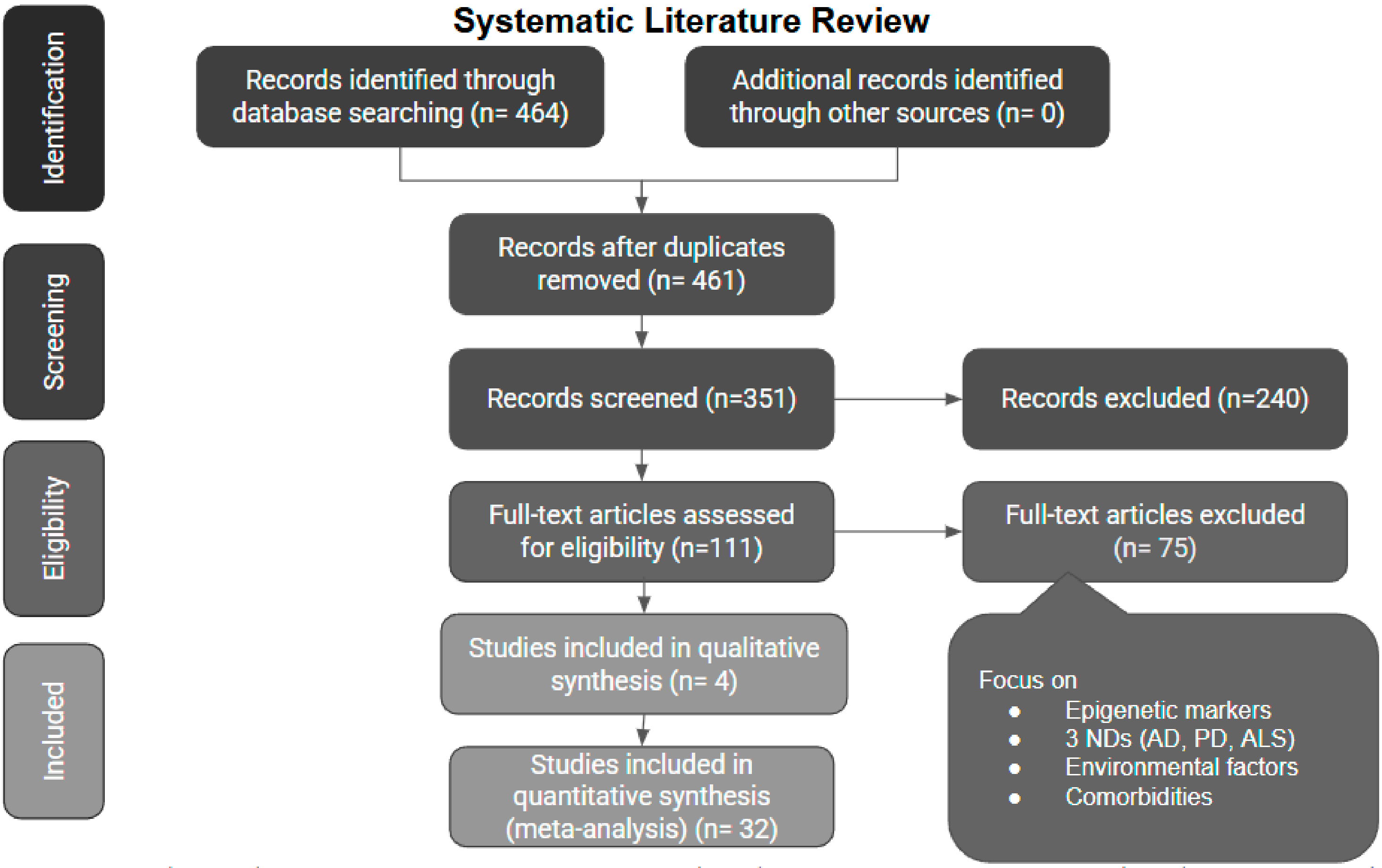

Supplemental Figure S2. PRISMA systematic literature review diagram of factors or comorbidities driving epigenetic marks associated with Alzheimer's Disease, Parkinson's Disease, or amyotrophic lateral sclerosis. Key words: epigen* AND (als OR (amyotrophic AND lateral AND sclerosis) OR (motor AND neuron AND disease) OR (lou AND gehrig's) OR parkinson's OR parkinsons OR parkinsonism OR alzheimer's OR alzheimers) AND (comorbidity or ((toxi* OR contaminant* OR poison*) AND ({exogenous} OR {external} OR {exposure}))))

3 Publication Trends.

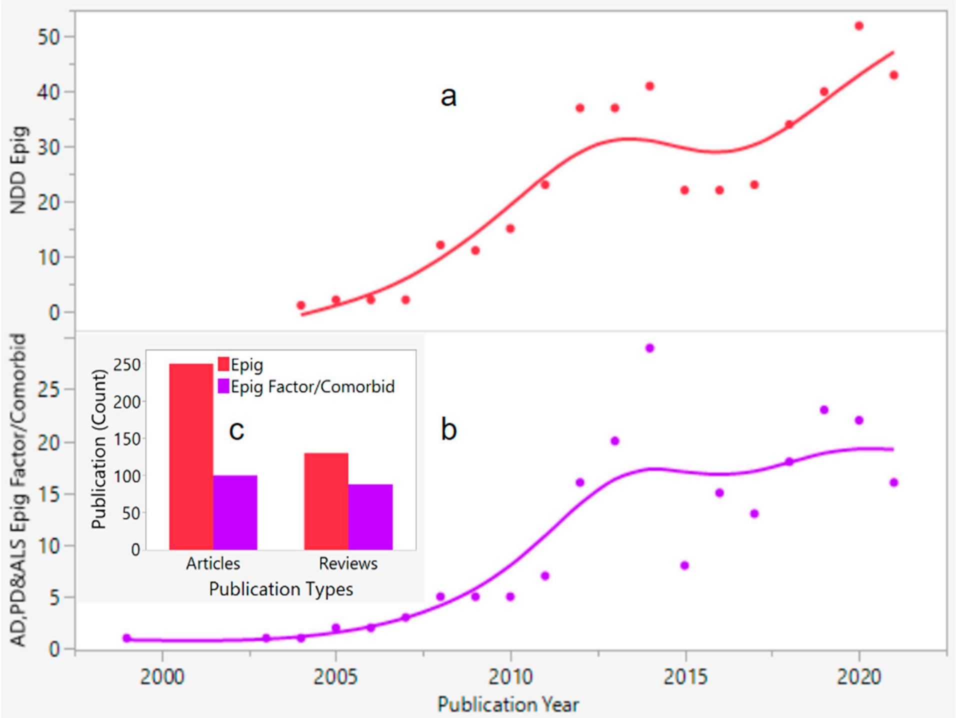

Supplemental Figure S3. Publication trends in epigenetics and factors/comorbidities associated with epigenetics in neurodegenerative disease PRISMA searches. Line graphs showing publications (count) over time of (a) epigenetic marks in neurodegenerative diseases and (b) factors/comorbidities driving epigenetic marks in AD, PD, and ALS. (c) Article:review ratios in factors/comorbidities associated with epigenetic marks in neurodegenerative diseases are shown in a histogram.

Publications of epigenetic marks associated with neurodegenerative diseases and specific factors or comorbidities driving epigenetic marks in AD, PD, and ALS increased since the 2000s, but plateaued prior to 2015 (Supplemental Figure S3). Research into epigenetic marks associated with neurodegenerative diseases has since increased. Analysis of article:review ratios has shown a relatively higher ratio in the wider search for epigenetic marks in neurodegenerative diseases (1.9:1) compared to factors or comorbidities associated with epigenetic marks in AD, PD, and ALS specifically (1.1:1). Nevertheless, the ratio of two primary research papers for every one literature review is still low and indicates that epigenetic mark research into neurodegenerative diseases has received minimal novel research in recent years.

4 Study locations for neurodegenerative disease**-associated toxins driving epigenetic.**

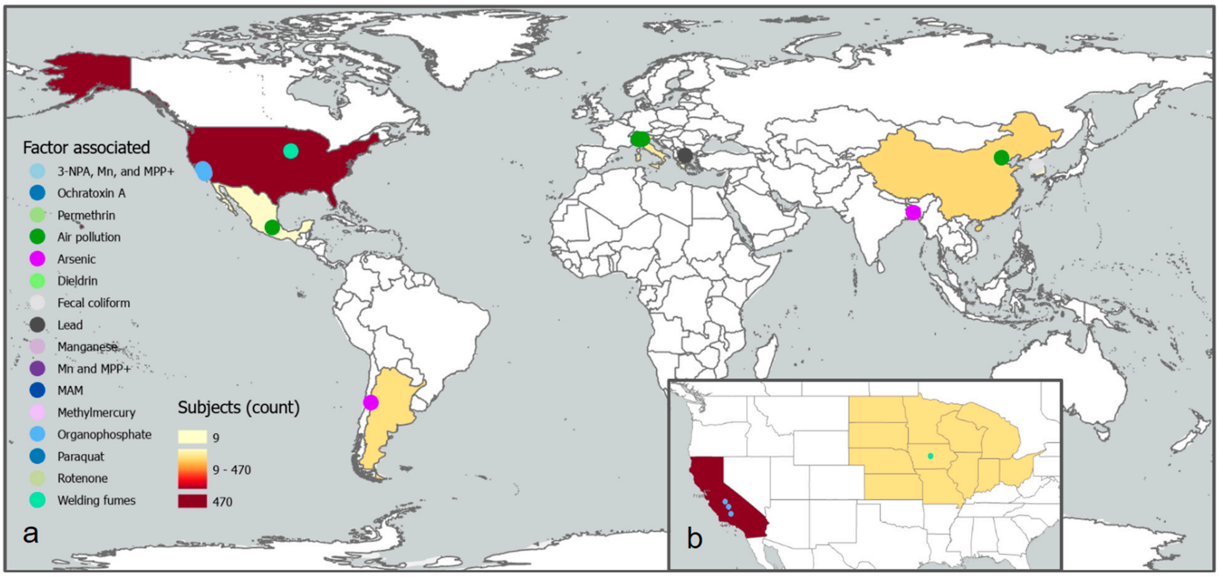

Figure S4. Location and subject count of studies associating exposure of environmental toxins with Alzheimer's Disease, Parkinson's Disease, and amyotrophic lateral sclerosis. (a) Environmental factors exposed to cohorts* or populations are plotted by subject count geographically (n=13) in ArcGIS. (b) California and midwestern United States regions highlighted.
*All subjects are human studies when reported geographically except for *Daphnia pulex* in Bu-cheon-si, South Korea.

5 Prevalence rates.

Supplemental Table S1. AD, PD, and ALS Prevalence rates (per 100,000 persons) by US state

High Prevalence	AD*	PD**	ALS***	Low Prevalence	AD*	PD**	ALS***
**Rhode Island**	227.0	265.0	5.1	**Utah**	104.6	163.9	4.4
**Connecticut**	224.9	260.0	5.1	**Alaska**	116.3	161.7	4.4
**Pennsylvania**	219.0	276.9	5.1	**Colorado**	130.9	207.6	4.4
**Maine**	214.8	300.4	5.1	**Texas**	136.2	188.4	4.7
**Iowa**	208.6	264.8	5.5	**Georgia**	140.1	200.0	4.7
**Vermont**	208.6	288.2	5.1	**Idaho**	147.8	231.7	4.4
**Wisconsin**	205.7	259.6	5.5	**Washington**	156.0	233.3	4.4
**South Dakota**	201.6	254.2	5.5	**Nevada**	156.1	227.9	4.4

*Alzheimer's prevalence reported for 2020,[1] and adjusted for population with 2020 US Census Bureau national and state population estimates.
** Parkinson's prevalence for 2016 reported by the Parkinson's Foundation,[2] and adjusted for population with 2016 US Census Bureau national and state population estimates.
*** ALS prevalence rates by region reported by the CDC.[3]

Supplemental Table S2. Statistical analysis comparing neurodegenerative disease prevalence rates and toxins associated with Alzheimer's Disease, Parkinson's Disease, and/or ALS by state

Neurodegenerative Diseases Prevalence Rates	Potential Associated Factors	Likelihood Ratio ChiSquare Test	P value
**Alzheimer's Disease, Parkinson's Disease, and ALS (Shared)**	Arsenic<br>Cadmium<br>Manganese<br>Mercury	10.645	0.0138
**Alzheimer's Disease and Parkinson's Disease**	Beta-HCH<br>Chlorpyrifos<br>Dimethoate<br>Iron<br>Naptha<br>Paraquat<br>PCB-101	71.127	<0.0001
**Alzheimer's Disease and ALS**	Aluminum<br>Cobalt<br>Copper<br>Lead<br>Selenium<br>Zinc	44.105	<0.0001
**Parkinson's Disease and ALS**	Magnesium<br>Methylene chloride<br>Nickel	6.099	0.0474
**Alzheimer's Disease, Parkinson's Disease, and ALS (Combined)**	Arsenic<br>Cadmium<br>Manganese<br>Mercury<br>Beta-HCH<br>Chlorpyrifos<br>Dimethoate<br>Iron<br>Naptha<br>Paraquat<br>PCB-101<br>Aluminum<br>Cobalt<br>Copper<br>Lead<br>Selenium<br>Zinc<br>Magnesium<br>Methylene chloride<br>Nickel	261.615	<0.0001

The Likelihood Ratio ChiSquare Test was used to determine if there is a correlation between neurodegenerative disease-associated toxins and prevalence rates. Significant p values are considered to be above a 95% Confidence Interval.

6 Additional Outstanding Questions.

An open question that results from this review is whether epigenetic marks associated with neurodegenerative diseases will be found in urine or feces of patients in early stages of disease. Cell apoptosis and other pathologies occurring in disease states are known to release cell-free DNA (cfDNA) with fragmentation patterns that may be unique to disease states.[4] More specifically, methylation status of cfDNA isolated from plasma of patients with AD has shown statistically significant differences in global hydroxymethylation compared to controls.[5] Epigenetic features in cfDNA could improve diagnosis by increasing the number of potential sites to monitor with low sample volumes and relatively low-cost methods,[4] including for neurodegenerative diseases. Furthermore, gastrointestinal changes in patients with PD,[6] opens the possibility that cells and cfDNA shed into feces with epigenetic changes in tow. Discovery of methylated status of cfDNA could be informed from flow cytometry or sequencing approaches, particularly in small fragments and when low detection limits are needed.[7] To date, the use of liquid biopsy, and specifically urine or feces, is not utilized in the suite of diagnostic tools implemented. However, if consistent differences in methylation patterns can be identified and viewed in cfDNA fragments extracted from liquid biopsy, methylation profiling could be used to identify various stages of neurodegenerative diseases. Clinical application of non-invasive methods for neurodegenerative disease diagnosis in individuals could benefit current screening participation.

If epigenetic marks are found in excreta, then wastewater-based epidemiology (WBE) may be used as another non-invasive tool to monitor public health indicators at the population level. Through isolation and concentration methodology, evaluating the waste products in WWTP influent may contribute to the measurement of population health markers. However, whether the methylation patterns will remain unchanged as the genomic DNA in cells or cfDNA fragments pass through the wastewater infrastructure to a collection site remains an open question. Furthermore, while heavy metal,[8] and pesticide,[9] analysis has been successfully applied to WBE to indicate regional exposure, methods to successfully extract methylated DNA from wastewater have yet to be established. Wastewater surveillance will offer a proactive means to coordinate a population-based screening program for the purpose of diagnosis earlier in the progression of disease.

References

1. Alzheimer's Foundation. Alzheimer's disease facts and figures [Internet]. Alzheimer's Association, 2023. [Cited 2022] Available from: https://www.alz.org/alzheimers-dementia/facts-figures

2. Marras C, Beck JC, Bower JH, Roberts E, Ritz B, Ross GW, et al. Prevalence of Parkinson's disease across North America. npj Parkinson's Disease. 2018 Jul 10;4(1):1–7.

3. Mehta P. Prevalence of Amyotrophic Lateral Sclerosis — United States, 2015. MMWR Morb Mortal Wkly Rep [Internet]. 2018 [cited 2022 May 24];67. Available from: https://www.cdc.gov/mmwr/volumes/67/wr/mm6746a1.htm

4. Angeles AK, Janke F, Bauer S, Christopoulos P, Riediger AL, Sültmann H. Liquid biopsies beyond mutation calling: Genomic and epigenomic features of cell-free dna in cancer. Cancers. 2021;13(22).

5. Chen L, Shen Q, Xu S, Yu H, Pei S, Zhang Y, et al. 5-Hydroxymethylcytosine Signatures in Circulating Cell-Free DNA as Diagnostic Biomarkers for Late-Onset Alzheimer's Disease. Journal of Alzheimer's Disease. 2022;85(2):569–81.

6. Warnecke T, Schäfer KH, Claus I, Del Tredici K, Jost WH. Gastrointestinal involvement in Parkinson's disease: pathophysiology, diagnosis, and management. npj Parkinson's Disease. 2022;8(1).

7. Li M, Chen WD, Papadopoulos N, Goodman SN, Bjerregaard NC, Laurberg S, et al. Sensitive digital quantification of DNA methylation in clinical samples. Nature Biotechnology. 2009;27(9):858–63.

8. Markosian C, Mirzoyan N. Wastewater-based epidemiology as a novel assessment approach for population-level metal exposure. Science of the Total Environment. 2019;689:1125–32.

9. Rousis NI, Gracia-Lor E, Hernández F, Poretti F, Santos MM, Zuccato E, et al. Wastewater-based epidemiology as a novel tool to evaluate human exposure to pesticides: Triazines and organophosphates as case studies. Science of The Total Environment. 2021 Nov 1;793:148618.

7 Review Sources Metadata

Supplemental Table S3. Factors and Comorbidities Driving Epigenetic Marks Associated with Alzheimer’s Disease, Parkinson’s Disease, and Amyotrophic Lateral Sclerosis

No.	Disease Associated	Cells / Tissue	Isolation / Extraction	Biomarker	Gene Function	Epigenetic Marker(s) Associated	Epigenetic Assay	Expression Mechanisms	Expression Mechanisms Assay	Factor Associated	Comorbidity	Model	Dose	Time	Association	Association Details	Subjects	Controls	Geographic Region	Source	Comment
1	Parkinson's	neuronal cells	nuclear pellet, acid extraction	global acetylation and Histone H3 and H4		Histone acetylation	Western Blotting<br><br>Chromatin Immunoprecipitation-sequencing (ChIP-seq)			3-nitropropionic acid, manganese (Mn) and 1-methyl-4-phenyl-pyridinium (MPP+)		N27 rat neuronal cells	0, 0.5, 1, 2, 4 mM	0, 2, 6, 18, 24, 48 h	differential acetylation	Acetylated histone H3K56 --> 1747 up-regulated and 263 down-regulated genes<br><br>dose/time effect: H3 and H4acetylation increase linearly, but H4 acetylation peaked at 6 h and decreased at 48 h			N/A	Ranganayaki et al., 2020	
2	(neurological)	blood	Wizard Genomic DNA Purification Kit	global 5mC and 5hmC		DNA methylation	Quest 5hmCTM DNA ELISA Kit 5mC DNA ELISA Kit			air pollution				4, 7, 14 days	hypermethylation	PM10 --> increases in 5hmC of 26.1% in office workers (P = 0.004), 20.2% in truck drivers (P = 0.014), and 21.9% in all participants combined (P < 0.001); PM10 effects on 5hmC were increasingly stronger when averaged over 4, 7, and 14 d preceding assessment (up to 132.6% for the 14-d average in all participants, P < 0.001). PM10 --> no	60 truck drivers 60 office workers		Beijing, China	Sanchez-Guerra et al., 2015	

																correlations with 5mC					
3	Alzheimer's	frontal white matter		H3K9me2 H3K9me3		histone post-translational modification (HPTM)	nuclear staining<br><br>immunostaining immunohistochemistry protocol immunofluorescence protocol			air pollution			high pollution-exposure area: above USA EPA and WHO PM10/PM2.5 standards control locations: below standards		increase active marks	H3K9me2 nuclear staining: decrease in global intensity of nuclei staining immunostaining: increase in "light" stained nuclei<br><br>H3K9me3 nuclear staining: decrease in global intensity of nuclei staining immunostaining: increase in "light" stained nuclei, and decrease in "medium" staining<br><br>pollution --> loss of repressive marks, gain of active epigenetic marks	13	10	Mexico	Calderón-Garcidueñas et al., 2020	urban
3	neuromuscular disease	buffy coats (white blood cells and platelets)		mtDNA: transfer RNA phenylalanine (MT-TF), 12S ribosomal RNA (MT-RNR1) gene and “D-loop” control region		DNA methylation	EZ DNA methylation Gold kit<br><br>bisulfite-Pyrosequencing			air pollution			Study 1: Particulate Matter with aerodynamic diameter >1 µm, high exposure: PM1 ≥ 7.6 µg/m3 low exposure: PM1 ≤ 3.8 µg/m3<br><br>Study 2: high exposure: personal airborne benzene	samplers during work day	differential methylation	PM1 exposure --> MT-TF and MT-RNR1 methylation (P = 0.025)<br>no association with air benzene or EC	60	60	Study 1: Brescia, Italy	Byun et al., 2013	Study 1: steel workers Study 2: gas-station attendants Study 3: truck drivers<br><br>Study 1: metal-rich PB<br>Study 2: air benzene<br>Study 3: traffic-derived Elemental Carbon (EC)

													≥ 31.2 μg/m3 low exposure: personal airborne benzene ≤ 23.0 μg/m3<br><br>Study 3: higher exposure: indivudal EC ≥ 16.6 μg/m3 lower exposure: individual EC ≤ 16.1 μg/m3								
3	neuromuscular disease	buffy coats (white blood cells and platelets)		mtDNA: transfer RNA phenylalanine (MT-TF), 12S ribosomal RNA (MT-RNR1) gene and “D-loop” control region		DNA methylation	EZ DNA methylation Gold kit<br><br>bisulfite-Pyrosequencing			air pollution			Study 1: Particulate Matter with aerodynamic diameter >1 μm, high exposure: PM1 ≥ 7.6 μg/m3 low exposure: PM1 ≤ 3.8 μg/m3<br><br>Study 2: high exposure: personal airborne benzene ≥ 31.2 μg/m3 low exposure: personal airborne benzene ≤ 23.0 μg/m3<br><br>Study 3: higher exposure: indivudal EC ≥ 16.6 μg/m3	samplers during work day	differential methylation	PM1 exposure --> MT-TF and MT-RNR1 methylation (P = 0.025)<br>no association with air benzene or EC	60	60	Study 2: Milan, Italy	Byun et al., 2013	Study 1: steel workers Study 2: gas-station attendants Study 3: truck drivers<br><br>Study 1: metal-rich PB Study 2: air benzene Study 3: traffic-derived Elemental Carbon (EC)

													lower exposure: individual EC ≤ 16.1 µg/m3								
4	neuromuscular disease	buffy coats (white blood cells and platelets)		mtDNA: transfer RNA phenylalanine (MT-TF), 12S ribosomal RNA (MT-RNR1) gene and “D-loop” control region		DNA methylation	EZ DNA methylation Gold kit<br><br>bisulfite-Pyrosequencing			air pollution			Study 1: Particulate Matter with aerodynamic diameter >1 µm, high exposure: PM1 ≥ 7.6 µg/m3 low exposure: PM1 ≤ 3.8 µg/m3<br><br>Study 2: high exposure: personal airborne benzene ≥ 31.2 µg/m3 low exposure: personal airborne benzene ≤ 23.0 µg/m3<br><br>Study 3: higher exposure: indivudal EC ≥ 16.6 µg/m3 lower exposure: individual EC ≤ 16.1 µg/m3	samplers during work day	differential methylation	PM1 exposure --> MT-TF and MT-RNR1 methylation (P = 0.025)<br>no association with air benzene or EC	60	60	Study 3: Beijing, China	Byun et al., 2013	Study 1: steel workers Study 2: gas-station attendants Study 3: truck drivers<br><br>Study 1: metal-rich PB<br>Study 2: air benzene<br>Study 3: traffic-derived Elemental Carbon (EC)
4	Alzheimer's	women: peripheral blood<br>new-borns: cord blood	DNA: QIAampH DNA Blood Midi kit<br><br>RNA: PAXgene Blood RNA kit<br><br>concentration analysis: NanoDrop	AS3MT	methylates inorganic arsenic for arsenic metabolism	DNA methylation	bisulfite treatment<br>EZ DNA Methylation kit<br><br>Infinium HumanMethylation450 BeadChip	RNA expression	Illumina DirectHyb HumanHT-12 v4.0	arsenic			women<br>n = 71: 200 mg/L in drinking water<br>n = 32: 7–73 mg/L in drinking water		hypermethylation	arsenic --> increased AS3MT methylation<br>altered methylation --> reduced expression of AS3MT	103	127	women: Argentinian Andes	Engström et al., 2013	exposure analysis: HPLC-ICPMS<br><br>103 women<br>127 new-borns

							pyrosequencing														
5	Alzheimer's	women: peripheral blood<br>new-borns: cord blood	DNA: QIAampH DNA Blood Midi kit<br><br>RNA: PAXgene Blood RNA kit<br><br>concentration analysis: NanoDrop	AS3MT	methylates inorganic arsenic for arsenic metabolism	DNA methylation	bisulfite treatment<br>EZ DNA Methylation kit<br><br>Infinium HumanMethylation450 BeadChip pyrosequencing	RNA expression	Illumina DirectHyb HumanHT-12 v4.0	arsenic			women<br>n = 71: 200 mg/L in drinking water<br>n = 32: 7–73 mg/L in drinking water		hypermethylation	arsenic --> increased AS3MT methylation altered methylation --> reduced expression of AS3MT	103	127	new-borns: Bangladesh	Engström et al., 2013	exposure analysis: HPLC-ICPMS<br><br>103 women 127 new-borns
6	Alzheimer's	cortex, hippocampus		H3K9: open chromatin state		Histone acetylation	Chromatin immunoprecipitation (ChIP) ab10812 antibody against acetylated H3K9<br><br>RT-qPCR Power SYBR Green PCR Master mix<br><br>negative control: glucagon (Gcg) primer<br>positive control: tubulin (Tubb3) primer	proteins	Western Blot	arsenic		mouse	Sodium arsenite water: 100 mg/L	adult mice: 2 weeks prenatal: 1 week, and during gestation	hypoacetylation	arsenic --> global hypoacetylation			N/A	Cronican et al., 2013	
7	Parkinson's	midbrain	DNA: phenol : chloroform : isoamyl alcohol RNase A<br><br>RNA: RNeasy Lipid Tissue Mini Kit	global methylation<br><br>Nr4a2 and Lmx1b	central nervous system neuron development pathway, dopaminergic neuron development and maintenance	DNA methylation	bisulfite sequencing TrueMethyl kit	RNA expression	RNA sequencing Kapa Illumina Library Quantification qPCR assays	dieldrin		C57BL/6 mice	0.3 mg/kg every 3 days	4 weeks	differential methylation	dieldrin --> differentially methylated Nr4a2 and Lmx1b genes	5	5	N/A	Kochmanski et al., 2019	organochlorine pesticide
8	Parkinson's	mesencephalic dopaminergic neuronal cells	histone extraction: NE-PER kit	H3 and H4		Histone acetylation	Western Blot anti-acetyl-Lys antibody			dieldrin		N27 rat neuronal cells<br><br>mouse	100uM. 10-30uM	1, 24 h	hyperacetylation	dieldrin --> hyperacetylation in H3 and H4 in 10 minutes<br><br>mouse model - 30 dayshyperacetylation in striatum and substantia nigra			N/A	Song et al., 2010	pesticide
9	Parkinson's		DNA extraction kit<br><br>concentration	global methylation		DNA methylation	MethylFlash global DNA methylation	proteins	high-pH reversed-phase peptide	fecal coliform		*Daphnia magna*			hypermethylation	contaminated stream water --> global	10	10	Bu-cheon-si,	Chatterjee et al., 2019	contaminated

			analysis: NanoDrop				5-mC ELISA Easy Kit		fractionation kits							hypermethylation			South Korea		stream water
10	Alzheimer's		Extraction: QIAamp kit			DNA methylation	methlyated DNA immunoprecipitation (MeDIP) protocol fluorescent images	RNA expression	NimbleGen 12 × 135K array	lead		mouse	0.2% Pb-acetate	in utero: gestational day 13 from drinking water exposure group: from mother's milk	hypermethylation	hypermethylated genes --> reduced gene expression methylation maintained later in life			N/A	Alashwal et al., 2012	C57Bl6 mice
10	Alzheimer's	neurons	Proteinase K protocol	global methylation		DNA methylation	neuron-specific genome-wide promoter DNA methylation Roche NimbleGen Mouse 3x720K CpG Island Promoter Array			lead		mouse: C57BL/6J background agouti viable yellow Avy strain	0, 2.1, 32ppm Pb-acetate water	2 weeks prior to mating	hypomethylation	lead --> hypomethylated 12 probes for high dose and 7 probes for low dose	6	3	N/A	Dou et al., 2019	Subjects: low: 3 high 3
10	Alzheimer's	blood	phenol–chloroform protocol	p16 promoter	tumor suppression, methylation represses suppression function	DNA methylation	bisulfite modification<br><br>PCR Platinum Taq polymerase 1.5% agarose gel			lead			7-100 μg/dL		hypermethylation	lead --> hypermethylated in high dose of lead	9	10	Thessaloniki, Greece	Leda at al., 2010	
11	Parkinson's	neuronal cells	RNA isolation TRIzol reagent	histone H3 and H4	acetylation/deacetylation regulates gene expression, chromatin remodeling, cell cylce progression, DNA repair, apoptosis	Histone acetylation	nuclear protein extract Cytoplasmic Extraction Kit<br><br>immunoblotting Western Blot	proteins	colorimetric HDAC activity assay kit<br><br>HAT activity colorimetric assay kit	manganese		rat pheochromocytoma cell line and PC12 cells<br><br>human SHSY5Y cells	300 μM MnCl2	3, 6, 12 and 24 h	hypoacetylation	manganese --> histone hypoacetylation, up-regulation of HDAC and down-regulation of HAT			N/A	Guo et al., 2018	
12	Parkinson's	neuronal cells	phenol:chloroform: isoamyl alcohol<br><br>concentration analysis: NanoDrop	PINK1, PARK2 and TH	neuronal development and differentiation, dopamine metabolic process, and ubiquitin-mediated proteolysis	DNA methylation	whole-genome bisulfite conversion EZ DNA Methylation Gold™ Kit<br><br>sequencing EpiGnome™ Methyl-Seq Kit EpiGnome polymerase		internal controls: RPII and HPRT	manganese		human SHSY5Y cells	100 μM MnCl2	30 days	differential methylation	manganese --> methylation of PINK1, PARK2 and TH			N/A	Tarale et al., 2017	
13	Parkinson's	dopaminergic neuronal cells		global methylation		DNA methylation	qPCR Eva-green ready mix Taq	Total RNA expression	microarray	manganese (Mn) and 1-methyl-4-phenyl-		N27 rat neuronal cells	Mn: 150 uM MPP+: 250 uM	Mn: 48 h MPP+: 48 h	differential methylation	Mn --> 529 methylated genes exclusively;			N/A	Mythri et al., 2017	MPP+ is a known neurotoxin

							Polymerase normalized: GAPDH<br><br>methylation array normalization: LOWESS<br><br>bisulfite treatment<br>EZ DNA methylation-Gold Kit<br><br>genome-wide pyrosequencing			pyridinium (MPP+)						194 hypermethylated genes, 209 hypomethylated genes<br>MPP+ --> 500 methylated genes exclusively, 174 hypermethylated genes, 211 hypomethylated genes					
14	ALS/Parkinson-dementia complex	medial prefrontal cortex (mPFC)	ProteoExtract Subcellular Proteome Extraction kit	H3 lysine K4 and K9		Histone methylation	Western Blot			methylazoxymethanol (MAM)	schizophrenia	rat	22 mg/kg/ml to pregnant moms		differential methylation	MAM prenatal --> postnatal methylation changes of H3			N/A	Maćkowiak et al., 2014	similar to BMAA
15	neurodegeneration	rat embryonic cortical stem cells	GeneElute Mammalian Genomic DNA Miniprep Kit<br><br>concentration analysis: NanoDrop	global methylation		DNA methylation	microplate reader MethylFlash Methylated DNA Quantification Kit	senescence markers	immunocytochemistry qPCR	methylmercury (MeHg)	neuro-developmental disorders	rat	2.5 or 5.0nM MeHg	48 h	hypomethylation	methylmercury --> decrease in global methylation			N/A	Bose et al., 2012	
16	neurotoxicity	cerebrum	DNeasy kits<br><br>concentration analysis: NanoDrop	global methylation		DNA methylation	LUminometric Methylation Assay (LUMA)			methylmercury (MeHg)		mink, chicken and yellow perch	mink: 0, 0.1, 0.5, 1, and 2 mg/kg nominal concentrations in diet		hypomethylation	methylmercury --><br>mink: reduced global DNA methylation in 1ppm dose but NOT 2ppm dose<br>no effect in other model animals			N/A	Basu et al., 2013	
17	neurotoxicity	sperm	Protinese K protocol<br><br>concentration analysis: NanoDrop	global methylation		DNA methylation	Methylated DNA Immunoprecipitation (MeDIP)<br>Qubit ssDNA Assay Kit<br><br>NEBNext1 Ultra™ RNA Library Prep Kit Illumina			methylmercury (MeHg)		zebrafish	0, 1, 3, 10, 30, and 100 nM nominal concentration as methylmercury chloride—CH3ClHg	<1 to 24 h	differential methylation	methylmercury --> increased epimutations in associated genes in the neuroactive ligand-receptor interaction and actin-cytoskeleton pathways			N/A	Carvan et al., 2017	
18	Alzheimer's and Parkinson's	kidney	TIANamp Genomic DNA kit	E-cadherin and N-cadherin		DNA methylation	bisulfite treatment<br><br>methylation			Ochratoxin A	nephrotoxicity	F344 rat	0. 70, 210 ug/kg body weight	4, 13, 26 weeks (5	hypermethylation	Ochratoxin A --> hypermethylation after 13	12	6	N/A	Li et al., 2015	Subjects:<br>6: low<br>6: high

				global methylation			specific PCR HotStart Taq Polymerase<br><br>HPLC-MS/MS							days/week)		week exposure					
19	Parkinson's	blood, saliva		global methylation		DNA methylation	genome-wide methylation assay Infinium HumanMethylation450 BeadChip			organophosphate					differential methylation	organophospate --> 7 differentially methylalted genes	470	369	Kern County, California	Paul et al., 2018	pesticide<br><br>Subjects: blood: 342 saliva: 128<br><br>Control: blood: 238 saliva: 131
20	Parkinson's	blood, saliva		global methylation		DNA methylation	genome-wide methylation assay Infinium HumanMethylation450 BeadChip			organophosphate					differential methylation	organophospate --> 7 differentially methylalted genes	470	369	Fresno County, California	Paul et al., 2018	pesticide<br><br>Subjects: blood: 342 saliva: 128<br><br>Control: blood: 238 saliva: 131
21	Parkinson's	blood, saliva		global methylation		DNA methylation	genome-wide methylation assay Infinium HumanMethylation450 BeadChip			organophosphate					differential methylation	organophospate --> 7 differentially methylalted genes	470	369	Tulare County, California	Paul et al., 2018	pesticide<br><br>Subjects: blood: 342 saliva: 128<br><br>Control: blood: 238 saliva: 131
22	Parkinson's	neuronal cells	histone extraction: NE-PER kit	H3 and H4		Histone acetylation	Western Blot anti-acetyl-Lys antibody			paraquat		N27 rat neuronal cells	400 μM	12, 24 or 36 h	differential acetylation	paraquat --> acetylation of H3			N/A	Song et al., 2011	agrochemical
23	Parkinson's	brain striatum	RNA Extraction: Aurum Total RNA Fatty and Fibrous Tissue Kit<br><br>Chromatin Extraction: cross-linked with 1% formaldehyde, glycine, PBS phenol/chloroform	Snca promoter	dopamine-synthesis pathway	chromatin fold enrichment<br><br>methylation	ChIP-qPCR<br><br>normalizing: β-actin<br><br>5mC DNA ELISA Kit Quest 5hmC DNA ELISA Kit<br><br>EpiJET Bisulfite	RNA expression	RT-PCR<br><br>normalizing: β-actin	Permethrin		rat	34.05 mg/kg dissolved in corn oil<br><br>(NOAEL 25 mg/kg)		hypermethylation	toxin increase--> increased global methylated cytocines	13	13	N/A	Bordoni et al., 2019	pyrethroids, pesticide Wistar rat

			Genomic DNA Extraction: DNAzol<br><br>GeneJET Genomic DNA Purification Kit				Conversion Kit<br><br>PyroMark PCR Kit pyrosequencing														
24	Parkinson's	human embryonic kidney cell HEK293	cell viability analysis: trypan blue (0.4%) staining<br><br>RNA: trizol method<br><br>DNA: Phenol:Chloroform :Isoamyl alcohol	HCN2 and NEFM	HCN2 - regulate neuronal plasticity<br>NEFM - support axonal growth and transport	DNA methylation	bisulfite treatment Sigma DNA Imprint Modifcation kit<br><br>2% agarose gel, ethidium bromide mini-Elute gel extraction kit<br><br>Ilumina Nextera DNA Library Preparation kit	RNA expression	RNA-sequencing TruSeq Sample Preparation kit 2% agarose gel miniElute gel extraction kit	rotenone		human cells	treatment: 200 nM rotenone control: 200 nM DMSO	24 h	hypomethylation	rotenone --> hypomethylation; differential RNA expression			N/A	Freeman et al., 2020	pesticide
25	Parkinson's	liver	Trizol reagent<br><br>concentration analysis: NanoDrop	global methylation		DNA methylation	whole-genome bisulfite sequencing	RNA expression	RNA-seq	rotenone		C57BL6/J mice	diet: 0, 10 or 150 ppm rotenone ad libitum	6, 12, 18 months old	hypomethylation	perinatal rotenone exposure --> Hypomethylation of the Avy Locus and global differential methylation in the Offspring	212		N/A	Lozoya et al., 2020	rotenone concentration analysis: HPLC
26	Parkinsonism	blood	QIAamp DNA blood kit	NOS2	regulating inducible nitric oxide synthase (iNOS), hypermethylation reduces producing of nitric oxide and hypomethylation increases production of nitric oxide (pro-inflammation mediator)	DNA methylation	bisulfite conversion Qiagen's EpiTect Fast DNA Bisulfite Kit<br><br>Pyromark PCR Master Mix agarose gel			welding fumes				less than 10 years of cumulative exposure	hypomethylation	welding fume --> reduced methylation in CpG site 8329 located in an exonic splicing enhancer 0.16% drop in methylation per year	98	103	Midwestern United States	Searles Nielsen et al., 2015	Subjects: 49 intermediate 49 parkinsonism

Supplemental Table S4. Epigenetic Marks Associated with Neurodegenerative Diseases

No.	Neurodegenerative Disease(s) Referenced	Tissue to Cell Pathway (For biomarkers)	Mechanisms Affected	Biomarkers Found	Specific Epigenetic Changes	Purpose or Utility of Biomarker	Patient Specimen # Detail	Controls # Detal	Analysis Techniques	Tissue Type	Source
1	Alzheimer's	N/A	N/A	N/A	17,895 differentially methylated CpG sites covering 8,678 genes identified. Total of 11,822 CpGs were hypermethylated and 6,073 CpGs were hypomethylated	N/A	34 total specimens, late onset AD	34 specimens with no dementia	Genome wide methylation data obtained from GEO database, used Illumina Infinium Human Methylation 450 array platform; tissue samples from STG region for all 68 samples; preprocessing procedure from Hyunh et al. Probes were removed from the data according to the following criteria: SNPs within 5 bp upstream of targeted CpG; minimum allelic frequency of <0.05; probes on X and Y chromosomes; cross-hybridising probes. Total of 424,497 CpGs were selected and used for subsequent analysis. Remaining CpGs were quantile normalized using the lumi package in R software and beta-mixture quantile method.	Superior temporal gyrus	Gao et al., (2017)
2	Parkinson's	N/A	Nucleic acid binding, Parkin expression	fosB proto-oncogene; functions along the mesocorticolimbic projection in the reward circuitry and along the nigrostriatal pathway affecting motor function	31 differentially methylated regions. Thirteen regions comprised of CpG sites that were hypermethylated in PD, and 18 regions were hypomethylated. The DMRs were found in 13 chromosomes; chromosome 6 alone contained nine DMRs		15 patients clinically diagnosed with PD; peripheral blood samples collected	15 normal, healthy individuals, peripheral blood samples collected	Isolated samples collected via PAXgene DNA/RNA isolation kits, stored at -20 C; examined methylation using Illumina Infinium Human Methylation 450K Beadchips; stained via Illumina Tecan system, fluorescence intensity data analyzed; mRNA was sequenced using the Illumina TruSeq RNA Library Prep kit	Whole blood DNA	Henderson et al., (2021)
3	Parkinson's	N/A	Only vaguely references brain specific alterations	LY6G5C, CYP2E1, Doublecortin Like Kinase 1, Dynein Cytoplasmic 1 Heavy Chain 1, LIM Domain Only 3, neurotransmission regulator synaptotagmin 12 (SYT12: p = 2.79E-7			380 participants from the Harvard Biomarker Study, 189 patients with PD	191 control subjects	RT-PCR	Whole blood	Henderson-Smith et al., (2019)
4	Alzheimer's	N/A	N/A	BACE1 (upregulated), Ncstn, Sirt1 ADORA2A	Increased acetylation in 15-month old mice, downregulated genes, Ncstn and Sirt1, the promoters were significantly less accessible in both or at least in one of the affected brain areas, BACE1 gene altered in PBMCs of AD patients		31 patients with AD	16 controls	RNA extracted, and cDNA obtained by reverse transcription reaction, then processed by rt-PCR	PBMCs	Marques et al., 2012
5	Alzheimer's	N/A	FAAH increase contributes to excitotoxic damage	FAAH protein (?) or FAAH mRNA	Alterations in FAAH mRNA, decreased methylation at FAAH gene, inverse relationship between methylation and gene expression		LOAD patients, 33 for DNA methylation studies and 12 for gene expression analysis	Equal # of age-controlled individuals with no cognitive disorders	PBMCs were separated by density gradient using the Lympho- lyte-H kit; RNA separated by standard procedures; q-RTPCR for analysis; methylation analysis by fluorescence real time PCR; Analysis of proteins by Western blot		D'Addario et al., 2012
6	Alzheimer's		Differentiation, proliferation, and survival of microglia; hematopoietic cell lineage, phagosome, Cytokine–cytokine receptor interaction, and chemokine signaling	N/A	478 and 187 significant DMRs associated with AD Braak stage; 119 co- methylated DMRs at 5% FDR; 118 out of the 119 DMRs included FDR significant individual CpGs. 421 out of the 3751 FDR significant		1030 prefrontal cortex brain samples + premortem		DMRs ID'd by coMethDMR	Prefrontal cortex (at Braak stage)	Zhang et al., (2020)

			pathways; inflammatory response, immune cell differentiation, and cytokine production,		individual CpGs overlapped with the FDR significant DMRs; majority of the significant methylation differences were hyper-methylated in AD, for which methylation levels were increased as AD stage increased. 58.6% of significant CpGs and 73.9% of significant DMRs were hyper-methylated in AD; significant hypermethy- lated DMRs and CpGs were both enriched in flanking active promoter regions (TssAFlnk), enhancers (Enh), Transcr. at gene 5′ and 3′ (TxFlnk), and polycomb repressed regions (ReprPC), but under-represented in promoter regions (TssA), strongly transcribed regions (Tx), and repressed regions; hypomethylated DMRs and CpGs were enriched in repressed regions (Quies, ReprPCWk) and weakly transcribed regions (TxWk), but under-represented in promoter regions (TssA, TssBiv)		whole blood samples				
7	Alzheimer's	Extrinsic apoptotic signaling pathway via death domain receptors; Negative regulation of single stranded viral RNA replication via double stranded DNA intermediate	Apoptosis pathway, protelolysis, oxoreductase activity	mir-20a-5p, mir-93-5p, mir-16- 5p, let-7b-5p, mir-708-5p, mir-24-3p, mir-26b-5p, mir-17-5p, mir-193- 3p, mir-186-5p	gene-set enrichment analysis showed that the DEGs were enriched in different biological processes, molecular functions and cellular components. The molecular pathway enrichment analysis revealed that pathways in ribosome related pathways, alternative and classical complement pathway and lectin induced complement pathway were altered		17 blood samples from AD patients	23 control samples	TarBase and miRTarBase to identify regulatory miRNAs; human histone modification database (HHMD) to find histone modification sites; uminescent signal was detected with a Zenyth ELISA reader	Peripheral blood mononuclear cells	Rahman et al., (2020)
8	Alzheimer's	N/A	Inflammatory cytokine release	MIP-2 (significant increase)	significant increase of monocytic H4K12 acetylation in both transgenic AD mouse models early during development of the plaque deposition in the brain. In line with these data we found significantly elevated acetylation of H4K12 in human patients with MCI but not in patients with AD. Further we observed that the monocytes of AD mice and of AD patients were significantly more vulnerable to cell damage and displayed an enhanced release of pro-inflammatory cytokines		34 patients with AD	31 people, not cognitively impaired	H4K12 measurement or FACS analysis or culture overnight; analyzed using the EpiQuik Global Acetyl Histone H4K12 Quantification Fluorometric Kit	Peripheral Monocytes	Plagg et al., (2015)
9	Alzheimer's		Lipid hydroperoxidation	5-Lipoxygenase, leukotriene B4	Hypomethylation of Alox5 promoter lead to increased levels of LOX-5 protein expression		27 Late Onset AD patients	28 healthy controls	RT-PCR of genes of the three main LOX isozymes: Alox5, Alox12, and Alox15, protein levels analyzed by Western Blot	Peripheral blood mononuclear cells	Francesco et al., 2013

10	Alzheimer's			Neurofilament (NF)-labeled pyramidal neurons that are vulnerable to AD pathology are deficient in extranuclear 5mC in AD cases compared with controls. We also found that fewer astrocytes exhibited nuclear 5mC and 5hmC marks in AD cases compared with controls	No significant difference in the percentage of NF-rich pyramidal neurons that colocalized with nuclear 5mC la- beling in layers 2/3 or 5 across control; significantly less NF-positive pyramidal neurons contained extranuclear 5mC label-ing in layer 5 in late-AD cases (14.8%, 1.3%) compared with control and early-AD cases (28.8%, 3.0%, 31.2%, 4.1%, respectively; Kurskal-Wallis 1-way ANOVA on ranks, p 0.05; significantly higher percentage of calretinin immunopositive interneurons with nuclear 5hmC in control, early-AD, and late-AD cases in layer 2/3 compared with layer 4 (1-way ANOVA, all p 0.001); ignificantly higher percentage of NF-positive pyramidal neurons exhibited nuclear 5mC in control cases compared with calretinin-positive interneurons in layer 2/3 (Mann-Whitney rank sum t test, p 0.05). Additionally, a signifi-cantly higher proportion of NF-positive pyramidal neurons contained nuclear 5hmC compared with calretinin-positive interneurons in layer 2/3 of control cases (t test, p 0.001). The percentage of NF-rich pyramidal neurons with extranuclear 5mC in layer 2/3 was significantly greater than that of calretinin-positive interneurons in control cases (Mann-Whitney rank-sum t test, p 0.05), whereas the proportion of NF-positive pyramidal neurons colocalized with extranuclear 5hmC in layer 2/3 was also signifi- cantly higher than that of calretinin-positive interneurons in control cases (Mann-Whitney rank-sum t test, p 0.05).		5 early AD cases, 6-8 late AD cases	5 healthy control samples	Immunohistochemistry performed with primary antibodies to assess astrocytes, microglia, calretinin-positive interneurons, NF-positive pyramidal neurons, NFTs, and Ab plaques in relation to 5mC and 5hmC marks, AB plaques immunolabelled	Postmortem tissue from cerebral cortex and inferior temporal gyrus	Phipps et al., (2016)
11	Parkinson's			A2AR mRNA	Consistent reduction in DNA methylation at gene promoter and an increase in histone H3 acetylation at lysine 9	Antagonize dopamine D2 receptor-mediated transmission	73 outpatients with diagnosis of sporadic PD, no signs of dementia	73 age and sex-matched controls	EpiSwitch was used on blood samples in a three-step process to identify, evaluate, and validate statistically-significant differences in chromo- somal conformations between ALS patients and healthy controls	PBMCs	Falconi at al., (2019)
12	Parkinson's		Stability of axonal cytoskeleton, indicated as a susceptibility gene in other neurodegenerative conditions such as progressive supranuclear palsy; MAPT mutations are a cause of monogenic frontotemporal dementia	MAPT-AS1 RNA, DNMT1, DNMT3A and DNMT3B	Significant decrease in MAPT-AS1 expression in PD ($p = 7.154 \times 10{-}6$); Knock-down expression of MAPT-AS1 led to a 1.3 to 6.3 fold increase in methylation of the endogenous MAPT promoter ($p < 0.011$) and a 1.2 to 1.5 fold increased expression of the 4-repeat MAPT isoform transcript	Amyloidogenic processing of APP Protein and activity increased in AD brains and CSF Critical for c-secretase stabilization; Overexpression increases Ab production Polymorphisms can increase AD risk	10 patients with diagnosed PD	10 healthy controls, no cognitive impairment	Genomic DNA and RNA were extracted from brain tissue using DNeasy and RNAQuick kits; PCR to amplify MAPT promoter region; Repeat Measure Mixed Linear Model Regression analysis, with a random intercept model, was used to explore the relationship between transcript levels and disease status across four brain regions.	Brain tissue samples from putamen, anterior cingulate cortex, visual cortex and cerebellum	Coupland et al., (2016)

						Overexpression reduces c-secretase in vivo and protects against Ab toxicity in vitro. Lower SIRT1 expression in AD brains; A2AR inhibition prevents Ab-induced neurotoxicity in vitro and in vivo					
13	Alzheimer's			140 unique disregulated miRNAs			Blood samples of 48 patients with AD	22 healthy controls	High-throughput NGS, WMW test, RT-qPCR		Leidinger et. al. (2013)
14	Alzheimer's			miR-137, -181c, -9, -29a/b	Downregulation of miR-137, -181c, -9, -29a/b	Regulation of ceramides	7 AD patient samples	7 healthy controls	Total RNA was quantified using ND-1000 nanodrop spectrophotometer; rt-PCR, products run on agarose gels	Blood serum samples	Geekiyanage et al., (2012)
15	Parkinson's		Ubiquitin proteasome system and glycosphingolipid biosynthesis	9 SNPs in USP37, SNP in ST8SIA4			19 patients with idiopathic PD	13 controls	Exiqon-developed miRCURYTM LNA microarrays, qRT-PCR	Blood mononuclear cells	Martins et. al. (2011)
16	Parkinson's						15 patients	15 controls	Examined methylation using Illumina Infinium HumanMethylation450K Beadchips; mRNA was sequenced using the Illumina TruSeq RNA Library Prep kit on the HiSeq 2000 platform; Expression quantitative trait methylation (eQTM) analysis was performed by applying the principle of expression QTL (eQTL) analysis to our DNA methylation and RNAseq datasets. Associations between differentially methylated loci and changes in gene expression were identified using the MatrixEQTL package	Whole blood (peripheral)	Henderson et. al
17	Alzheimer's		Cell cycle entry, tau phosphorylation; facilitate increases in both amyloid-beta protein precursor and beta-site cleaving enzyme (BACE1) in AD brain; oxidative stress and DNA damage, generated in part by elevations in amyloid-beta peptide, iron, and reactive oxygen species, would induce expression of miR-34a and miR- 146a	miR-9, miR-29a, miR-29b, miR-34a, miR-125b, and miR-146a	Plasma miR-34a and miR-146a levels, and CSF miR-34a, miR-125b, and miR-146a levels in AD patients were significantly lower than in control subjects. On the other hand, CSF miR-29a and miR-29b levels were significantly higher than in control subjects		10 patients	10 controls	RT-PCR	Plasma, CSF	Kiko et. al. (2013)
18	Alzheimer's		Phosphatidylinos- itol binding clathrin assembly		PICALM methylation was decreased in AD compared to HS (mean = 3.54 and 4.63, respectively, p = 0.007). In AD, PICALM methylation level was also positively associated to Mini-Mental Scale Examination (MMSE) score (percent change 3.48%, p = 0.008). Moreover, a negative association between PICALM methylation and age was observed only in HS (percent change –2.29%, p = 0.002)		43 AD subjects	38 healthy patients	Quantitative bisulfite-PCR pyrosequencing	Whole blood	Mercorio et. al. 2018

19	Alzheimer's and Parkinson's		Braak stage, dementia status, plaque and tangle densities, and the presence and severity of Lewy body pathology	miR-184 and miR-127-3p	Differential regulation of around 41 different miRNAs		69 AD, 67 PD patients	78 control samples	miRNA expression profiling using NGS	CSF and SER samples	Burgos et. al. (2014)
20	Alzheimer's		Cell cycle progression, transcription factor binding		Total of 17,895 differentially methylated CpG sites were initially identified, including 11,822 hypermethylated CpGs and 6,073 hypomethylated CpGs	Hypomethylated genes affected transcription factor binding, hypermethylated genes corresponded to cell cycle progression	34 patients with late onset AD	34 patients without dementia	Data obtained from Gene Expression Omnibus database; methylation values for individual CpGs in each sample were expressed as β-values, compared against controls using t-test; hierarchical clustering analysis	Tissue samples from STG region	Gao et. al. (2017)
